# Novel AAV-mediated genome editing therapy improves health and survival in a mouse model of methylmalonic acidemia

**DOI:** 10.1371/journal.pone.0274774

**Published:** 2022-09-20

**Authors:** Shengwen Zhang, Amy Bastille, Susana Gordo, Nikhil Ramesh, Jenisha Vora, Elizabeth McCarthy, Xiaohan Zhang, Dylan Frank, Chih-Wei Ko, Carmen Wu, Noel Walsh, Shreya Amarwani, Jing Liao, Qiang Xiong, Lauren Drouin, Matthias Hebben, Kyle Chiang, B. Nelson Chau

**Affiliations:** LogicBio Therapeutics Inc., Lexington, MA, United States of America; Fudan University, CHINA

## Abstract

Methylmalonic acidemia (MMA) is an inborn error of metabolism mostly caused by mutations in the mitochondrial methylmalonyl-CoA mutase gene (*MMUT*). MMA patients suffer from frequent episodes of metabolic decompensation, which can be life threatening. To mimic both the dietary restrictions and metabolic decompensation seen in MMA patients, we developed a novel protein-controlled diet regimen in a *Mmut* deficient mouse model of MMA and demonstrated the therapeutic benefit of mLB-001, a nuclease-free, promoterless recombinant AAV GeneRide^TM^ vector designed to insert the mouse *Mmut* into the endogenous *albumin* locus via homologous recombination. A single intravenous administration of mLB-001 to neonatal or adult MMA mice prevented body weight loss and mortality when challenged with a high protein diet. The edited hepatocytes expressed functional MMUT protein and expanded over time in the *Mmut* deficient mice, suggesting a selective growth advantage over the diseased cells. In mice with a humanized liver, treatment with a human homolog of mLB-001 resulted in site-specific genome editing and transgene expression in the transplanted human hepatocytes. Taken together, these findings support the development of hLB-001 that is currently in clinical trials in pediatric patients with severe forms of MMA.

## Introduction

MMA is a metabolic disorder most commonly caused by mutations in the methylmalonyl-CoA mutase (*MMUT*) gene. MMUT deficiency leads to the failure of converting methylmalonyl-CoA to succinyl-CoA in the propionate pathway, with a subsequent accumulation of toxic metabolites such as methylmalonic acid in tissues and circulation [1, 2]. The severity of symptoms among the patients with *MMUT* mutations is correlated with the type of mutation and the amount of residual enzyme activity retained. Patients can have a complete loss of enzyme activity, designated as Mut^0^, or a partial reduction, designated as Mut^-^. The Mut^0^ subtype is associated with high morbidity and mortality at an early age [1], while Mut^-^ patients tend to be more heterogeneous and symptoms present later with lower morbidity and mortality rates [2].

Patients with MMUT deficiency are treated with strict life-long dietary management to mitigate acute illness that worsens the patient’s basal condition, particularly with respect to metabolic brain injury [3]. Restoring hepatic methylmalonyl-CoA mutase activity through liver transplantation can improve metabolic stability, reducing the incidence of metabolic decompensation, often normalizing renal function and stabilizing neurocognitive function [4, 5]. However, liver transplantation is an invasive, high-risk procedure, with the potential of transplanted organ failure and life-long comorbidities. Restoration of MMUT enzymatic activity by gene therapy mediated by recombinant adeno-associated virus (rAAV) is a promising approach [6–9]. However, rapid hepatic growth during the neonatal/pediatric period results in dilution of the episomal DNA and consequently loss of therapeutic efficacy [10]. Similarly, diseases with high hepatic turnover limit the benefit of episomal-driven gene therapies. Genome editing would thus be the best approach for pediatric patients and for diseases with rapid cellular turnover, allowing the phenotypic correction of the edited cells to be passed on to the daughter cells. However, endonuclease-mediated gene editing approaches carry the risks of immunogenicity of the endonucleases, off-target cleavage, and mutagenesis [11]. The gene editing GeneRide^TM^ technology leverages the sequence-guided endogenous process of homologous recombination to insert a promoterless therapeutic gene into a specific genomic locus without the aid of nucleases, resulting in the expression of the transgene driven by an endogenous promoter [12].

LB-001 is a GeneRide AAV vector designed to provide durable therapeutic hepatic expression of MMUT protein. The expression of MMUT is driven by the hepatocyte-specific *albumin* (*ALB*) promoter following site-specific integration (S1 Fig). The vector genome contains a copy of the *MMUT* coding sequence preceded by a porcine 2A peptide sequence [13], flanked by two 1-kb homology sequences corresponding to the *ALB* locus flanking the stop codon. Site-specific integration of the vector genome occurs precisely following, and in-frame with, the penultimate codon of the *ALB* gene. The transcription product is a fused *ALB-2A-MMUT* mRNA driven by the endogenous *ALB* promoter. Upon translation, the 2A sequence mediates ribosomal skipping, resulting in the synthesis of two separate proteins, a carboxyl-terminal 2A-modified albumin (ALB-2A) and MMUT. ALB-2A is secreted into the circulation and conveniently serves as a transgene expression biomarker that can be monitored over time as a surrogate for the therapeutic protein expression, negating the need of liver biopsy where the MMUT protein is shuttled to its native subcellular mitochondrial localization. The durability of gene editing suggests that LB-001 offers an opportunity to intervene at an early age before MMA patients succumb to the disease.

To provide preclinical support for the development of LB-001, a mouse surrogate, mLB-001, was constructed with a copy of the mouse *Mmut* coding sequence and homology sequences corresponding to the mouse *Alb* locus, and evaluated in a mouse model of MMA. The *Mmut*^*-/-*^*;Tg*^*INS-MCK-Mmut*^ mice (herein referred to as MMA mice or *Mmut*^*-/-*^) are systemically deficient in endogenous *Mmut*, while expressing it specifically in the muscle from a transgene driven by the muscle creatine kinase (MCK) promoter [14]. The mouse model recapitulates clinically relevant features of patients with severe MMA, including reduced survival, retarded growth, and elevated levels of organic acids in blood and urine. The heterozygous mice (*Mmut*^*+/-*^), with or without the *MCK-Mmut* transgene, have a normal phenotype like the wild-type animals. To mimic the patient experience more closely, we developed a novel low protein diet regimen to reflect the dietary restrictions used in MMA patients and applied a high protein diet challenge to induce metabolic crises similar to what patients suffer. The treatment of both neonatal and young adult MMA mice with mLB-001 demonstrated a therapeutic benefit and protection from metabolic decompensation.

## Materials and methods

### GeneRide vectors

The vector genome of mLB-001 contained a copy of the mouse *Mmut* coding sequence and homology sequences corresponding to the mouse *Alb* locus. The vector genome of Vt196 contained a copy of the human *MMUT* coding sequence and homology sequences corresponding to the human *ALB* locus. The AAV vectors were produced in HEK293 cells by transient transfection of plasmids containing the vector genome, cDNA sequences encoding capsid and other helper proteins, followed by AAVX-affinity purification and CsCl gradient purification. The titer was determined by ddPCR. The mLB-001 and Vt196 vectors employed capsids AAV-DJ [15] and AAV-LK03 [16], respectively. All AAV vector were produced internally.

### Animal experiments

This study was carried out in strict accordance with the recommendations in the Guide for the Care and Use of Laboratory Animals of the National Institutes of Health. All animal studies and procedures were approved by the Institutional Animal Care and Use Committee at Mispro Biotech Services Corporation (Protocol Numbers: LOGC-004, LOGC-063, LOGC-094, LOGC-130). All efforts were made to minimize animal suffering.

All animals were housed in plastic containers fitted to the Innorack^®^ IVC Mouse 3.5 racks. The containers were replaced with fresh bedding, nesting and enrichment materials (wood chips, paper strips and paper cellulose domes) every two weeks or more frequently as needed. The housing room temperature was maintained at 20°C to 26°C with a relative humidity level of 30% to 70%. Animals had access to food and water *ad libitum*. Food/water supply and animal welfare were checked daily. Euthanasia was carried out by CO_2_ exposure followed by cervical dislocation or cardiac puncture.

#### Mouse model of MMA

The *Mmut*^*-/-*^*;Tg*^*INS-MCK-Mmut*^ (MMA mice) had a mixed FvB/NJ and C57BL/6J background, deficient in endogenous *Mmut* and expressing an *Mmut* transgene driven by the muscle-specific creatine kinase promoter. The initial breeders were obtained from Dr. Charles Venditti at National Institutes of Health. Breeding of phenotypically normal heterozygous females with a recombinant AAV-*Mmut* vector-rescued homozygous males resulted in two types of MMA pups, the homozygotes with the transgene (*Mmut*^*-/-*^*;Tg*^*INS-MCK-Mmut*^) and homozygotes without the transgene (*Mmut*^*0*^). Litter sizes ranged from 4 to 11. Genotyping was performed for all live pups at PND 0 or 1. The *Mmut*^*0*^ mice usually died within a few days after birth [17], and their data are excluded from analysis. All MMA mice enrolled in the studies were genotype-confirmed *Mmut*^*-/-*^*;Tg*^*INS-MCK-Mmut*^ mice (designated as MMA mice or *Mmut*^*-/-*^). The healthy littermates *Mmut*^*+/-*^ (heterozygotes with or without *Tg*^*INS-MCK-Mmut*^) served as controls and housing companions. Due to the severe disease phenotype of the MMA mice, weaning occurred on PND 28, one week later than our usual practice with healthy mice. All surviving pups stayed with their own dams until weaning and non-enrolled mice (*Mmut*^*0*^ and excessive *Mmut*^*+/-*^) were euthanized after weaning. When groups were assigned later in life for diet treatment and adult dosing studies, gender and starting body weight of the MMA mice were balanced when possible. Animals were fed with standard rodent chow (21% protein content, LabDiet 5053), 12% protein chow (Envigo 93253) or 40% protein chow (Envigo 90018). All diets had similar fat and caloric contents. Mice were intravenously injected with vehicle or vectors via facial veins under ice-anesthesia (neonates) or via lateral tail veins (adults). Animals were monitored for survival daily and body weight measured approximately twice a month. Typical condition for the MMA mice includes hunched posture and prominent backbone. Euthanasia was performed for animals considered as moribund, displaying severe adverse signs including prostration, decreased motor activity, inability to right, cold to touch, pale, and/or tremors. Animals were sampled approximately monthly by submandibular bleed, and terminal blood and liver were collected at necropsies.

#### Chimeric mice with a humanized liver

The chimeric PXB mice with a humanized liver were purchased from PhoenixBio Co Ltd, who produced the mice by xenotransplanting human hepatocytes into immunodeficient recipient cDNA-uPA^+/-^/SCID mice. Adult male mice of 5-month-old were intravenously injected with a GeneRide vector via lateral tail veins.

#### Change of diet protein content

The objective of this experiment was to evaluate the effect of low protein diet on the survival of the MMA mice without any other treatment. A total of 8 litters were enrolled in the study. Newborn mice initially fed on mothers’ milk until approximately two weeks of age. To provide a complete nutrition to the pregnant and nursing dams, a standard rodent chow (21% protein diet) was used until PND 14. On PND 14, when the dietary management started, litters were randomly assigned to two groups, with diet maintained on standard chow or switched to a 12% protein diet. Pups were weaned on PND 28 and circulating methylmalonic acid levels were measured on PND 30. At 7 weeks of age, the surviving animals on the 12% protein diet were switched to 21% protein diet to study the impact of increased protein intake on animal survival.

#### Systemic injection of mLB-001 in neonatal MMA mice

The objective of this experiment was to evaluate the effect of the GeneRide vector mLB-001 on the disease phenotype of the MMA mice treated during the neonatal period. A total of 15 litters were enrolled in the study, and all MMA mice were genotype-confirmed *Mmut*^*-/-*^*;Tg*^*INS-MCK-Mmut*^ mice. On PND 1, litters were randomly assigned to vehicle or mLB-001 group and all pups from the same litter (genotypes unknown at the time) received the same treatment (either vehicle or 1×10^14^ vg/kg mLB-001). Nursing dams were on the standard 21% protein diet until PND 14, then the diet was switched to the 12% protein diet. Pups were weaned on PND 28. MMA mice were socially housed with at least one heterozygous animal. At 3 months post-dosing, all animals were switched first to the standard chow with 21% protein content and monitored for clinical signs, body weight, and mortality. At 4 months post-dosing, all animals were switched to a 40% protein diet and monitored for an additional 2 months through necropsy at 6 months of age. The dose-response study was conducted with the researchers blinded to the treatment groups and a total of 39 litters were enrolled.

#### Systemic injection of mLB-001 in adult MMA mice

The objective of this experiment was to evaluate the effect of the GeneRide vector mLB-001 on the disease phenotype of the MMA mice treated as adults. MMA mice from 4 litters maintained on the 12% protein diet were enrolled in the study. At 8 weeks of age, mice from each litter were divided into two groups with an attempt to balance gender and body weight (N = 7 per group). Animals were injected intravenously with vehicle or 5×10^13^ vg/kg mLB-001 via tail vein. Two and three randomly selected heterozygous littermates were dosed intravenously with vehicle and 5×10^13^ vg/kg of mLB-001, respectively, to serve as controls and to evaluate the baseline genomic DNA integration rate. At 3 months post-dosing (5 months of age), all animals were switched first to the 21% protein standard chow, and at 4 months post-dosing (6 months of age), all animals were switched to the 40% protein diet. The animals were monitored for an additional 2 months through necropsy at 6 months post-dosing (8 months of age).

#### Systemic injection of a human specific GeneRide vector in adult PXB mice with a humanized liver

The objective of this experiment was to evaluate the ability of a human specific GeneRide vector to edit the genome of human hepatocytes and to express the therapeutic protein MMUT in vivo. The PXB mice purchased from Phoenix Bio were randomly assigned to two groups (N = 4 per group). At 5 months of age, mice were intravenously injected with 5×10^13^ or 1×10^14^ vg/kg of the GeneRide vector Vt196 via tail vein and monitored for 8 weeks through necropsy.

### Plasma alanine aminotransferase activity assay

Plasma alanine aminotransferase activity was quantified using an alanine aminotransferase activity colorimetric assay kit (BioVision) with 1:10 diluted plasma samples.

### Circulating methylmalonic acid quantification

Circulating methylmalonic acid was quantified in whole blood or plasma samples using a qualified liquid chromatography-tandem mass spectrometry analysis (LC-MS/MS), performed by Nextcea Inc. Methyl-d3-MMA was used as an internal standard (IS). MMA and methyl-d3-MMA were derivatized with N-ethyl-N’-(3-dimethylaminopropyl) carbodiimide and 2,2,2-trifluroethylamine and quantitated by LC-MS/MS using a Shimadzu XR Ultra High Performance Liquid Chromatograph system and a SCIEX Triple Quad 6500 mass spectrometer.

### Plasma ALB-2A fusion protein and albumin quantitation

ALB-2A was quantitated in mouse plasma samples in an enzyme-linked immunosorbent assay (ELISA) using a proprietary recombinant monoclonal rabbit anti-2A antibody for capture and an HRP-labeled anti-ALB polyclonal antibody (Abcam) for detection. Purified recombinant mouse or human ALB-2A was used as standards to build calibration curves from which ALB-2A concentrations were interpolated. Plasma mouse and human albumin levels were measured in species-specific ELISA (Abcam).

### Targeted genomic DNA integration in liver

Genomic DNA was extracted from frozen liver tissues and targeted genomic DNA integration was analyzed by long-range polymerase chain reaction (PCR) amplification, followed by quantitative polymerase chain reaction (qPCR) quantification using a qualified method (S1 Fig). Long Range PCR was performed using a forward primer (F1) and a reverse primer (R1). The PCR product was purified with solid phase reversible immobilization beads (ABM, G950) and used as template for qPCR using the forward primer (F1), a reverse primer (R2) and a probe (P1). The primers and probes for mouse experiments are (F1m) 5’-ATGTTCCACGAAGAAGCCA-3’, (R1m) 5’-TCAGCAGGCTGAAATTGGT-3, (R2m) 5’-AGCTGTTTCTTACTCCATTCTCA-3’, (P1m) 5’-AGGCAACGTCATGGGTGTGACTTT-3’. The mouse transferrin receptor (*Tfrc*) was used as an internal control in qPCR. The primers and probes for humanized mouse experiments are (F1h) 5’-GCTCTCCTGCCTGTTCTTTAG-3’, (R1h) 5’-TCAGCAGGCTGAAATTGGT-3, (R2h) 5’-TCAGCATAATAAGGGCAACACT-3’, (P1h) 5’-GCAAGAACTGTCAATTCAAGCTAGCAACT-3’. The human RNA pyrophosphohydrolase (*RPPH*) was used as an internal control in qPCR.

### Fused mRNA in liver

Total RNA was extracted from frozen liver tissues and the transcript from the integrated transgene was quantified by reverse transcription-coupled droplet digital polymerase chain reaction (ddPCR) using a qualified method with a forward primer (F2), a reverse primer (R3) and a probe (P2) (S1 Fig). The primers and probes for mouse experiments are (F2m) 5’-CACACTTCCAGAGAAGGAGAAGC-3’, (R3m) 5’-TCAGCAGGCTGAAGTTGGT-3’, (P2m) 5’-AAGACGCCTTAGCCGGCAGCGGC-3’. The primers and probes for humanized mouse experiments are (F2h) 5’-TGAGAAGGAGAGACAAATCAAGAA-3’, (R3h) 5’-TCGCCGGCCTGTTTCAG-3, (P2h) 5’-TTAGGCTTAGGAAGCGGCGC-3’.

### MMUT protein expression in liver by immunoblot analysis

Whole liver tissue lysates were prepared with buffer containing 50 mM Tris-HCl, 150 mM NaCl and 0.5% NP40 (pH = 7.5). Mitochondrial fractions were prepared from tissue lysates containing 210 mM mannitol, 70 mM sucrose, 5 mM Tris-HCL and 1 mM ethylenediaminetetraacetic acid, pH = 7.5 following a published method [18]. Whole tissue lysates or mitochondrial fractions were subject to immunoblot analysis for MMUT protein expression using rabbit polyclonal anti-MMUT antibody (Abcam ab134956). Beta-actin and the mitochondrial protein cytochrome c served as sample loading controls.

### Protein expression in liver by immunohistochemistry

MMUT and Ki67 protein expression in liver tissues was analyzed in formalin-fixed, paraffin-embedded tissue sections of 5 μm by immunohistochemistry analysis using rabbit polyclonal anti-MMUT antibody (Proteintech 17034-1-AP) and rabbit monoclonal anti-Ki67 antibody (Abcam ab16667), respectively.

### MMUT mutase activity in liver

MMUT mutase activity was measured in terminal liver tissues using a modified protocol based on a published method [19]. Liver tissue homogenates containing 50 μg protein were subject to enzymatic activity analysis in the presence of 0.4 mM methylmalonyl-CoA. The holo enzyme activity was measured in the presence of 0.2 mM adenosylcobalamin. The converted product succinyl-CoA was quantified using LC-MS/MS by Nextcea Inc.

### Statistics

Raw data were recorded and calculated when appropriate using Microsoft Excel. Graphs were generated and statistical analyses performed using Prism version 9 (GraphPad). Data in texts and graphs represent means and standard deviations unless noted. One-way analysis of variance, mixed-effects analysis with multiple comparison or two-sided Student’s *t*-tests were performed to compare values between groups, where statistical significance was defined as *P <* 0.05.

## Results

We have previously shown that a single systemic administration of a GeneRide vector to MMA mice led to targeted genomic integration of the transgene into the mouse *Alb* locus, and that the edited hepatocytes repopulated the liver through selective advantage conferred by improved mitochondrial function and decreased oxidative stress in the corrected hepatocytes [20]. Consistent with recent reports that some MMA patients have underlying liver diseases, including neoplasms [21, 22], the liver enzyme alanine aminotransferase was found to be significantly elevated in the MMA mice (S2 Fig). Furthermore, when the liver tissues of the MMA mice were stained for the proliferation marker Ki67, we found that the number of Ki67-positive hepatocytes was significantly higher than in the healthy heterozygotes (S3 Fig), suggesting a high rate of hepatocyte turnover in the diseased liver. In mLB-001-treated MMA mice, this increased rate of hepatic regeneration provides an opportunity for the healthier, gene-edited MMUT-expressing hepatocytes to repopulate the liver faster than the unedited diseased cells, resulting in the selective advantage and expansion of corrected cells. Hepatic regeneration and expansion of edited hepatocytes were observed under all protein diet conditions tested in this study, suggesting that selective advantage of corrected hepatocytes could occur even with dietary management in MMA patients.

When fed a standard rodent diet, MMA mice displayed lower body weight than healthy littermates starting at 2 weeks of life. After weaning at PND 28, the animals gradually perished, with less than 15% surviving through 3 months of age (S4 Fig). This standard diet, however, does not reflect how MMA patients are managed clinically, and the short life span poses challenges to evaluating the long-term therapeutic efficacy of mLB-001 treatment. This led us to modify the animal model by reducing the dietary protein level mimicking clinical management of MMA patients and extending the lifespan of MMA mice to allow demonstration of the therapeutic benefit of mLB-001 on clinical outcomes and biochemical biomarkers.

### Development of a clinically relevant MMA mouse model

Newborn mice initially feed on mothers’ milk until approximately two weeks of age [23]. To provide complete nutrition to the pregnant and nursing dams, a standard rodent chow (21% protein diet) was used during this period. The dietary management started on PND 14 when litters were randomly assigned to two groups: one group was maintained on the standard chow; the other group was switched to a 12% (low) protein diet. The low protein content was chosen to mimic the levels in nursing dam’s milk [24]. Pups were weaned on PND 28 and circulating methylmalonic acid levels were measured in blood samples collected on PND 30. Body weight was measured weekly, and animal survival was monitored through 7 weeks of age.

**Fig 1:**
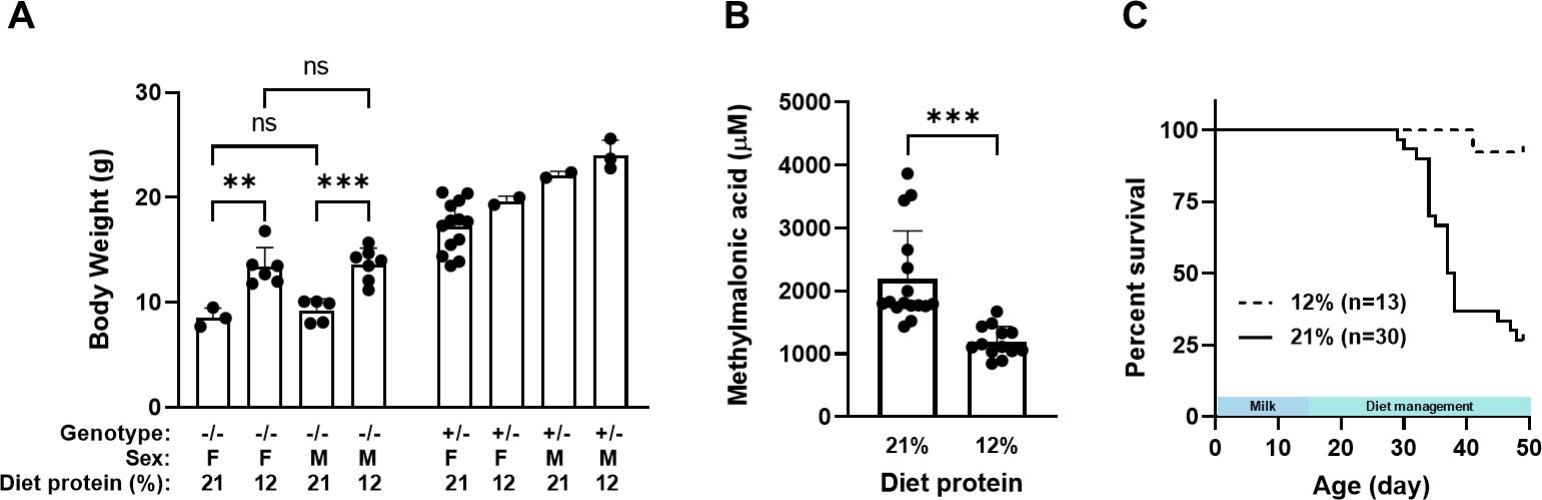
Body weight, circulating methylmalonic acid and animal survival in MMA mice on diets with standard (21%) and low (12%) protein content. Nursing dams were initially on a 21% protein diet. On PND 14, litters were either maintained on the 21% protein diet or switched to a 12% protein diet. (A) Body weight of MMA mice (-/-) and their heterozygous littermates (+/-) on PND 35. All heterozygous groups were significantly heavier than the gender- and diet-matched MMA groups. (B) Circulating methylmalonic acid level in MMA mice at PND 30. (C) Kaplan-Meier survival curves of MMA mice demonstrated significantly better survival of the 12% diet group than the 21% diet group. *** *P <* 0.001, **** *P <* 0.0001, Student’s *t*-test in (A) and (B). *P <* 0.0001, log-rank test in (C).

The low protein diet significantly improved the growth of MMA mice compared to the mice maintained on the 21% standard chow, even though the diet did not normalize the body weight, as shown at 5 weeks of age (Fig 1A). The levels of the circulating biomarker of the disease, methylmalonic acid, were significantly lower in MMA mice on the low protein diet than those on the 21% protein diet (Fig 1B), and the protein-restricted mice showed better survival (Fig 1C). Animals maintained on the 21% protein diet started to perish around weaning, and by 38 days of age, more than half of the mice succumbed to the disease. In contrast, over 90% of animals maintained on the 12% protein diet survived. These results suggest that the low protein diet offers significant protection to the MMA mice against post-weaning stress, reducing circulating methylmalonic acid levels, improving growth and reducing mortality. This protection resembles the clinical management of MMA patients maintained on protein-restricted diets.

To assess the impact of an early, temporary protein restriction on animal survival, the animals maintained on the 12% protein diet were returned to the standard 21% protein diet at 7 weeks of age and monitored for 3 additional months (managed group). These animals gradually died after the diet switch, with a median survival of 121 days (S5 Fig). This was a significant improvement compared to the unmanaged group fed only the 21% protein diet (*P <* 0.01, log-rank test). Nevertheless, these results demonstrate that a temporary low protein diet can offer only a transient benefit to the MMA mice.

### Systemic delivery of mLB-001 improved disease phenotype of MMA mice

#### Single dose of mLB-001 in neonatal MMA mice

We demonstrated that implementing a protein-restricted diet early in the animal model, as is done in patients with MMA, stabilized and extended the lifespan of the animals, protecting them from early death seen under the standard diet. We hypothesized that despite animals being stabilized, an increase in protein intake could trigger metabolic decompensation similar to what patients often experience and would allow evaluation of the therapeutic benefit of mLB-001 in a controlled manner.

**Fig 2:**
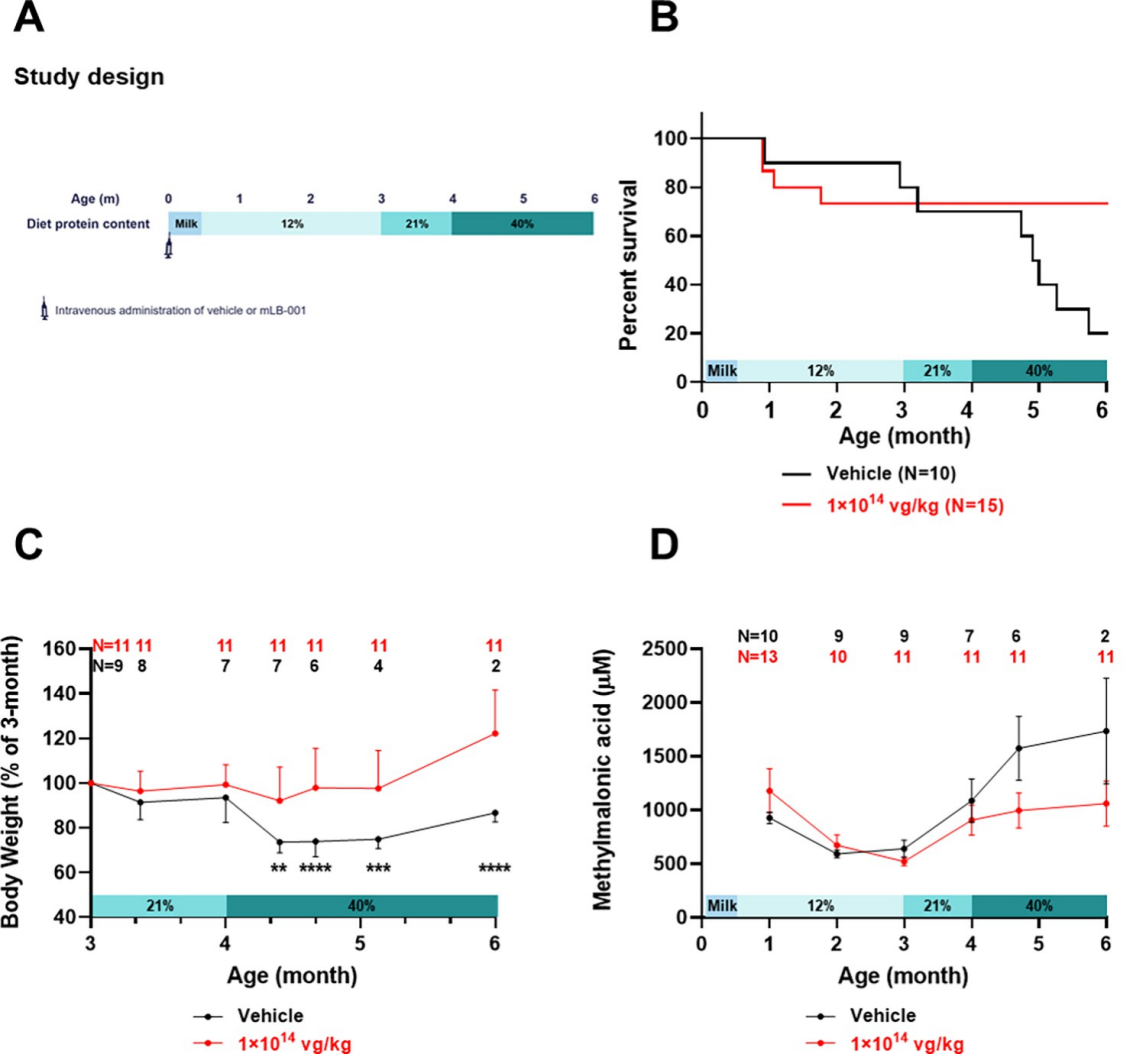
Effect of the high protein diet challenge on MMA mice treated with vehicle or 1×10^14^ vg/kg mLB-001 on PND 1. (A) Study design. Animals were dosed with vehicle or mLB-001 on PND 1. Nursing dams were kept on the standard 21% protein diet until PND 14, when the diet was switched to a 12% protein diet. Pups were weaned on PND 28 and maintained on the 12% protein diet. At 3 months of age, all surviving animals were switched to the standard chow with 21% protein content and then to a 40% protein diet at 4 months of age. Necropsy was performed at 6 months of age. (B) Kaplan-Meier survival curves of MMA mice. Log-rank test demonstrated significantly better survival of the mLB-001-treated group than the vehicle-treated group (*P <* 0.05). (C) Change in body weight of MMA mice in response to the high protein diet challenge. The body weight of each Mmut-deficient animal was normalized to its weight at the introduction of the 21% protein diet. Females and males were combined due to a lack of significant difference. Animal numbers at each timepoint are indicated above the graph. ** *P <* 0.01, *** *P <* 0.001, **** *P <* 0.0001, mixed-effects analysis with multiple comparisons between vehicle- and mLB-001-treated groups at indicated timepoints. (D) Circulating methylmalonic acid levels in MMA mice. The mLB-001-treated animals maintained lower levels than the vehicle-treated animals in response to the high protein diet challenge.

Neonatal mice, both *Mmut*^*-/-*^ and their healthy heterozygous littermates, were injected intravenously via facial vein with vehicle or 1×10^14^ vg/kg mLB-001 and maintained with the 12% protein diet. The initial rate of mLB-001-mediated homologous recombination resulted in <1% of edited hepatocytes, as measured in the heterozygous animals. Due to the high rate of hepatic turnover in the diseased MMA animals, we hypothesized that the gene-edited, MMUT-expressing healthy hepatocytes would repopulate the liver over time and achieve systemic metabolic stability, even without low protein dietary management. In contrast, the vehicle-treated animals were expected to perish due to the transient nature of the protection offered by the low protein diet alone. To test this hypothesis, we applied a stepwise increase in the protein content of the diet, from 12% to 21% (the standard chow), at 3 months post-treatment with mLB-001, and then to 40% at 4 months post-dosing (Fig 2A). During the first three months of life, under the 12% protein diet, there was a 20–25% mortality in both vehicle- and mLB-001-treated groups (Fig 2B). Upon imposing the high protein diet challenge, the vehicle-treated mice gradually perished with a median survival of 148 days (58 days after the introduction of the 21% protein diet), while there was no mortality in the mLB-001-treated group. Log-rank test demonstrated significantly better survival of the mLB-001-treated group than the vehicle-treated group in response to the high protein diet challenge (*P <* 0.05). Under the 12% protein diet, there was no statistically significant difference in animal body weight between vehicle- (V) and mLB-001- (T) treated MMA animals, or between females (F) and males (M) within each dosing group (VF 16.0±1.2 g (N = 4), VM 19.2±3.9 g (N = 5), TF 17.6±1.9 g (N = 7), TM 19.4±3.2 g (N = 4) at 3 months of age). However, in response to the high protein diet challenge, the vehicle-treated mice lost 20–30% of body weight, followed by the death of most animals, while mLB-001-treated mice maintained their body weight and survived the challenge (Fig 2B and 2C). The treatment was not able to restore the body weight of the MMA mice as they remained significantly smaller than their healthy heterozygotes (S6A Fig). We monitored the circulating methylmalonic acid levels over time. During the first three months of life, while on the 12% protein diet, the methylmalonic acid levels showed an age-dependent decrease in both vehicle and mLB-001-treated animals, with no statistically significant difference between the two groups. During the high protein diet challenge, methylmalonic acid levels increased in both groups. However, lower and stable levels were consistently measured in the mLB-001-treated animals compared to those of the vehicle-treated animals (Fig 2D), suggesting that mLB-001-treated MMA mice were able to efficiently process the increased metabolite flux induced by the high protein diet. Due to the gradual loss of animals in the vehicle group during the challenge, the number of survivors (N = 2 at the 6-month timepoint) was insufficiently powered for a valid statistical comparison with the mLB-001-treated group.

**Fig 3:**
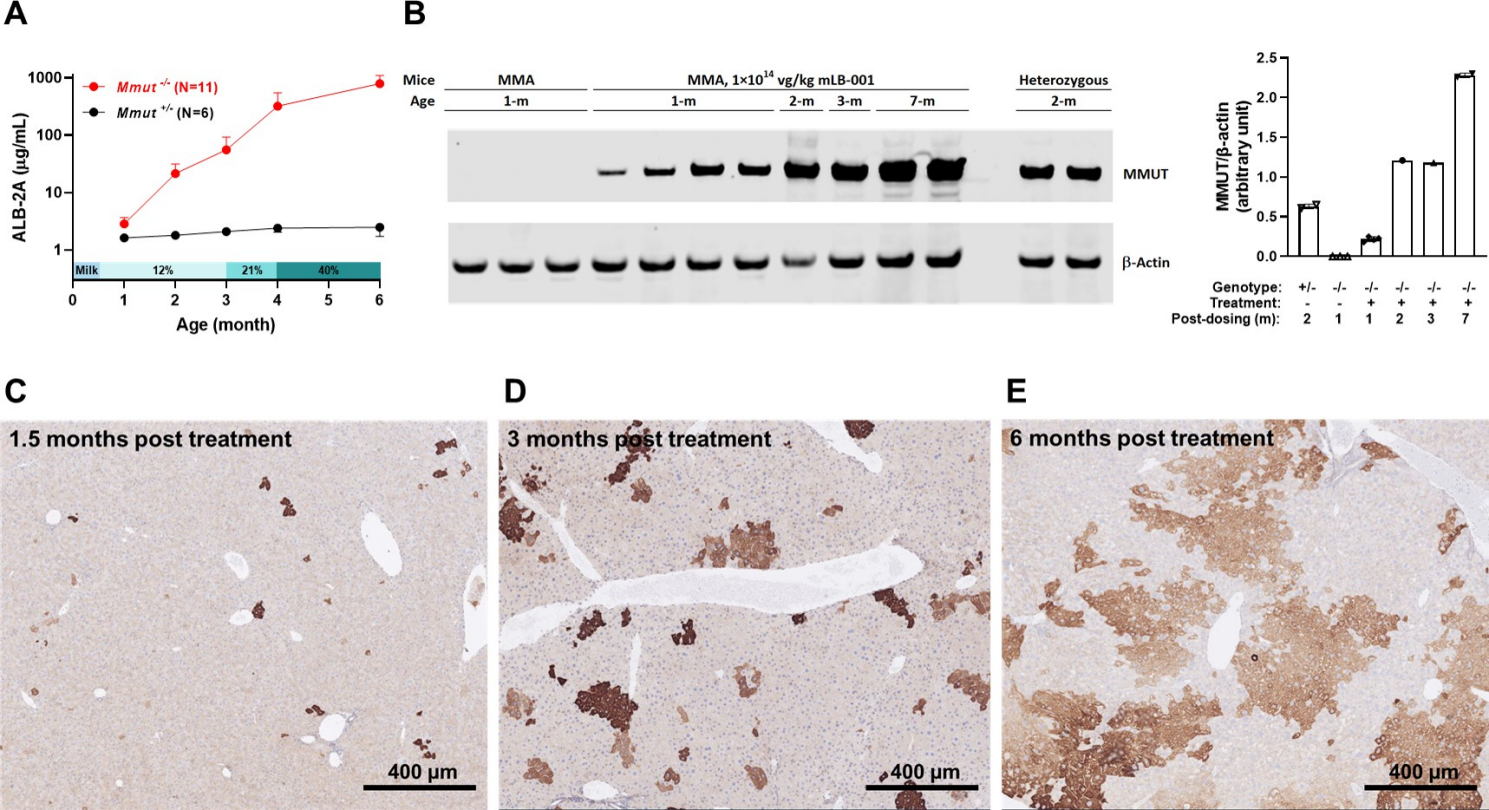
MMUT protein expression in mice treated with 1×10^14^ vg/kg mLB-001 at PND 1. (A) Plasma ALB-2A levels remained stable in all heterozygous (*Mmut*^*+/-*^) animals while showing an exponential increase over time in all MMA (*Mmut*^*-/-*^) mice. (B) Immunoblot analysis of MMUT protein in the liver tissues. Vehicle-treated heterozygous and MMA mice were used as positive and negative controls for protein expression. β-actin was used as a loading control. (C-E) Immunohistochemistry analysis of MMUT protein expression in livers of three mLB-001-treated MMA mice sacrificed at 1.5- (C), 3- (D), and 6- (E) months post-dosing. The scale bars represent 400 μm.

We monitored the GeneRide expression biomarker ALB-2A in circulation during the course of the study. We observed a progressive increase over time in the mLB-001-treated MMA mice while the level remained constant in the treated healthy heterozygous littermates (Fig 3A). Furthermore, the ALB-2A increase in the MMA mice was accompanied by an increasing amount of MMUT protein in liver lysates (Fig 3B) and increasing numbers of MMUT-expressing hepatocytes (Fig 3C–3E). Together, these data support the hypothesis that the edited, MMUT-expressing hepatocytes display a selective advantage in repopulating the liver of the diseased animals. In addition, the direct correlation between ALB-2A and MMUT protein expression validates the use of ALB-2A as a circulating pharmacodynamic biomarker for site-specific integration, transgene expression and hepatic selective advantage, supporting its use in clinical development. Improved survival, body weight and selective expansion of edited hepatocytes were confirmed in a separate dose-response study, with neonatal mice in randomized litters injected with vehicle (V) or three dose levels of mLB-001: 2.5×10^13^ (L), 5×10^13^ (M), and 1×10^14^ (H) vg/kg. The same aforementioned dietary management regimen was applied, and animals were followed with weekly clinical observation until the scheduled termination of the study at 6 months of age.

**Fig 4:**
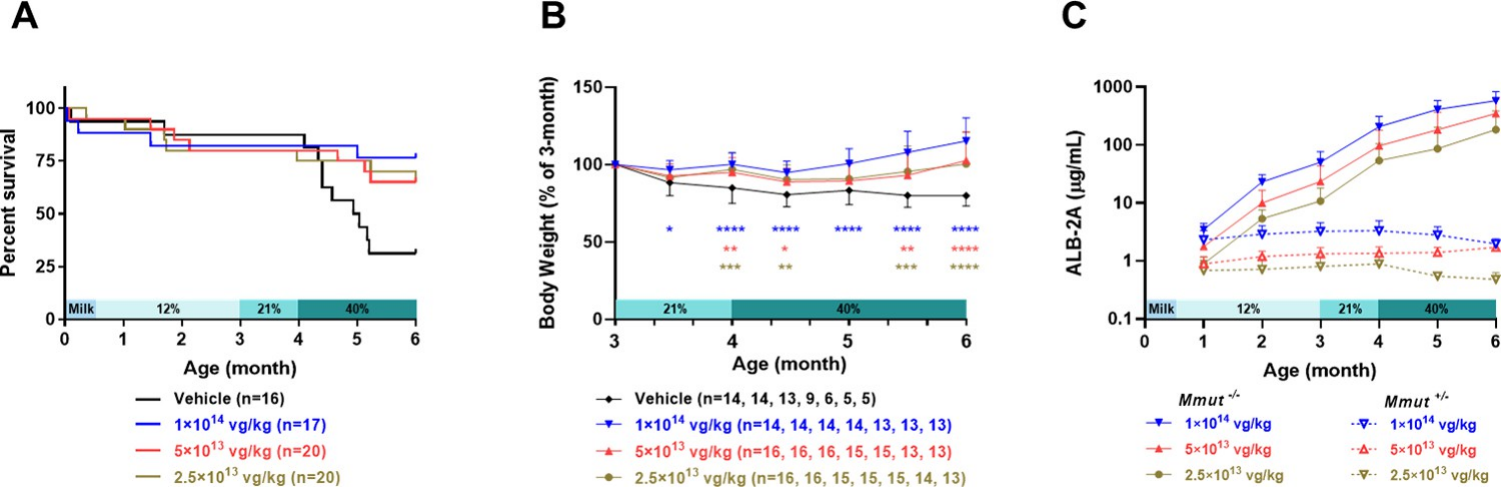
Effect of high protein diet challenge on MMA mice treated with vehicle or three dose levels of mLB-001 at PND 1. (A) Kaplan-Meier survival curves of MMA mice injected with vehicle or different doses of mLB-001. Log-rank test demonstrated significantly better survival of all three mLB-001-treated groups than the vehicle group during diet challenges (*P <* 0.05). (B) Change in body weight of MMA mice in response to the high protein diet challenge. The body weight of each Mmut-deficient animal was normalized to its weight at the introduction of the 21% protein diet. At all dose levels, mLB-001-treated animals demonstrated better maintenance in body weight than the vehicle-treated animals. Females and males were combined due to a lack of significant difference. Numbers in parentheses in the legends represent animal numbers at each timepoint. * *P <* 0.05, ** *P <* 0.01, *** *P <* 0.001, **** *P <* 0.0001, mixed-effects analysis with multiple comparisons between vehicle- and each mLB-001-treated group at the indicated timepoints. (C) Dose-dependent plasma ALB-2A levels demonstrated exponential increases over time in all mLB-001-treated MMA mice while remaining stable in all treated heterozygous animals. The rate of ALB-2A increase in the MMA mice was similar across all 3 treatment groups.

Consistent with the single-dose level study, 10–20% of the mice perished in all groups during the first three months of life. At the time of the diet challenge, at 3 months of age, the number of MMA animals in each group was 14 (V), 16 (L), 16 (M) and 14 (H). At the conclusion of the study at 6 months of age, the number of surviving animals in each group was 5 (V), 13 (L), 13 (M) and 13 (H). Log-rank test demonstrated significantly better survival of all three mLB-001-treated groups than the vehicle-treated group in response to the high protein diet challenges (*P <* 0.05) (Fig 4A). There was no significant difference in animal survival among the three mLB-001-treated groups. The body weight of the MMA mice in all three mLB-001-treated groups was well maintained during the high protein diet challenge, while the vehicle-treated group showed weight loss that failed to recover. The few surviving animals in the vehicle-treated group had significantly lower body weight than the mLB-001-treated animals (Fig 4B, S6B Fig). Circulating ALB-2A levels demonstrated time-dependent increases in the MMA mice while remaining stable in the healthy heterozygous littermates (Fig 4C), as seen in the single-dose study, supporting the expansion of the corrected hepatocytes in MMA mice. It is noted that the total albumin level (including the native and P2A-tagged forms) was comparable between vehicle- and mLB-001-treated groups and did not change over time (S7 Fig).

#### Single dose of mLB-001 in adult MMA mice

**Fig 5:**
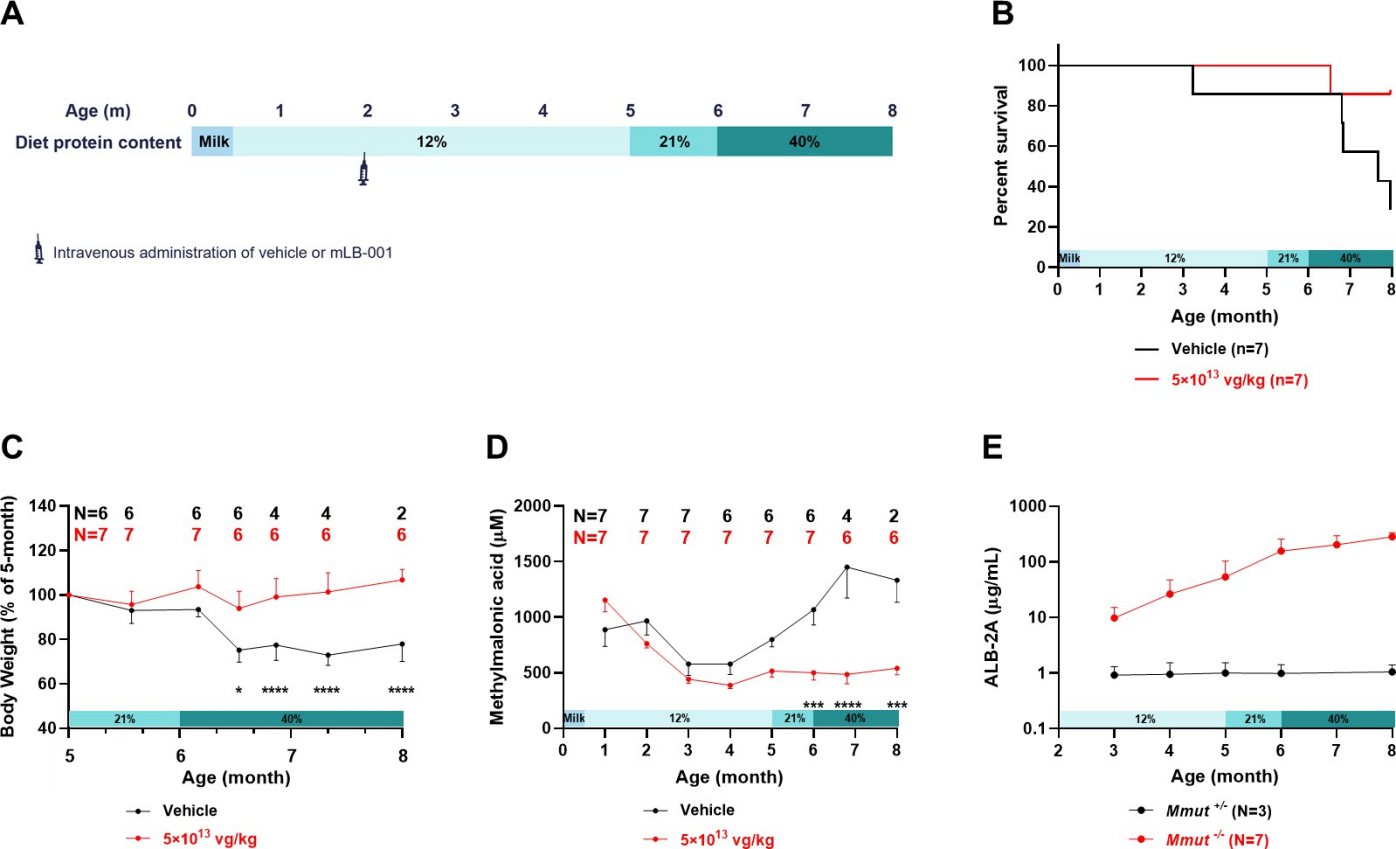
Effect of high protein diet challenge on MMA mice treated with vehicle or 5×10^13^ vg/kg mLB-001 at 8 weeks of age. (A) Study design. Animals were maintained on the 12% protein diet and dosed with vehicle or mLB-001 at 8 weeks of age. At 3 months post-dosing, the diet was switched to the standard chow with 21% protein content and then to a 40% protein diet at 4 months post-dosing. Necropsy was performed at 6 months post-dosing when the mice were approximately 8 months old. (B) Kaplan-Meier survival curves of MMA mice show that the mLB-001-treated group survived longer than the vehicle-treated group (*P* = 0.052, Log-rank test). (C) Change in body weight of MMA mice in response to the high protein diet challenge. The body weight of each animal was normalized to its weight at the introduction of the 21% protein diet. Females and males were combined due to lack of significant difference. Animal numbers at each timepoint are indicated above the graph. * *P <* 0.05, **** *P <* 0.0001, mixed-effects analysis with multiple comparisons between vehicle- and mLB-001-treated groups at the indicated timepoints. (D) Circulating methylmalonic acid in the mLB-001-treated animals maintained at low levels but elevated in the vehicle-treated animals in response to high protein diet challenge. *** *P <* 0.001, **** *P <* 0.0001, mixed-effects analysis with multiple comparisons between vehicle- and mLB-001-treated groups at the indicated timepoints. (E) Plasma ALB-2A levels remained stable in the mLB-001-treated heterozygous animals while demonstrating exponential increases over time in the MMA mice.

Improvements in the standard of care of MMA over the past few decades have allowed for many patients to survive to be young adults. However, these older patients still live with strict dietary management and experience unpredictable metabolic decompensations, and thus could benefit from durable gene therapies. To evaluate the therapeutic efficacy of mLB-001 in young adult animals, MMA mice maintained on the 12% protein diet were randomly assigned to a treatment group at 8 weeks of age and injected with either vehicle or 5×10^13^ vg/kg mLB-001. At 3 months post-dosing (5 months of age), animals were challenged with the same high protein diet regimen as previously described for neonates (Fig 5A).

Similar to the observations in neonatal dosing studies, five out of seven vehicle-treated MMA mice died in response to the high protein diet challenge, while only one out of seven in the mLB-001-treated MMA group died (Fig 5B). Clinical observation revealed that the mLB-001-treated mice were more active than the vehicle-treated animals, as shown by two representative animals in the video recorded at 5 months post-dosing (S1 Movie). The 40% protein diet challenge starting at 4 months post-dosing induced a 20–30% weight loss in the vehicle-treated animals within two weeks, from which they did not recover. In contrast, the mLB-001-treated animals maintained their body weight (Fig 5C, S6C Fig), although their body weight was not able to catch up to their gender-matched heterozygous littermates throughout the study. MMA mice showed an age-dependent reduction in circulating methylmalonic acid during the first few months of life when the disease was stabilized under the low protein diet. Upon challenge with the high protein diet starting at 5 months of age (3 months post-dosing), the mLB-001-treated animals maintained stable levels of methylmalonic acid while the vehicle-treated animals experienced significant increases in methylmalonic acid (Fig 5D). As seen previously in neonate dosing studies, circulating ALB-2A levels remained stable in the mLB-001-treated healthy heterozygotes but showed a time-dependent increase in the MMA mice dosed as adults (Fig 5E), indicating that a selective advantage of the edited hepatocytes occurred in the diseased, full-grown livers when the older, diet-managed MMA mice were treated under stabilized conditions. Immunohistochemistry analysis of livers at 6 months post-dosing revealed MMUT-expressing hepatocytes in patches similar to the neonatally dosed animals (S8 Fig), supporting the selective expansion of the GeneRide-edited hepatocytes.

### Molecular analyses of terminal samples

**Fig 6:**
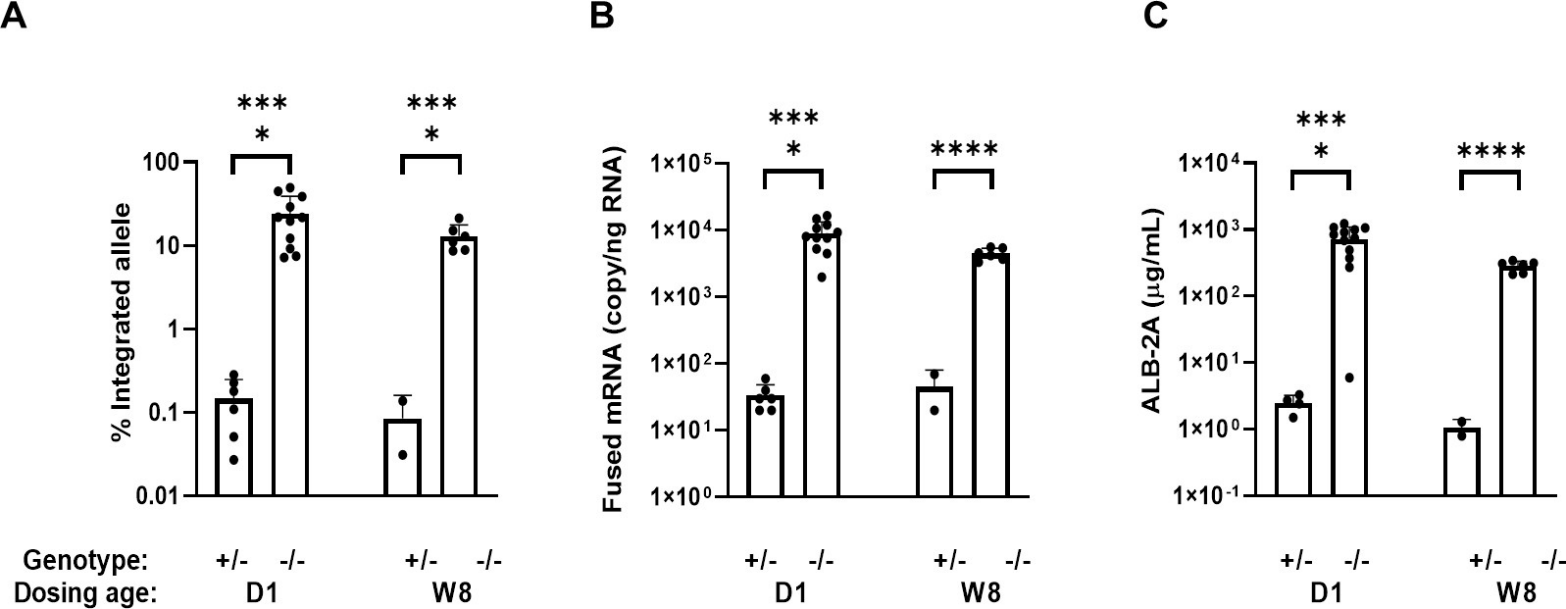
Analyses of terminal samples from animals treated with vehicle or mLB-001 at 6-month post-dosing. MMA mice (-/-) or heterozygous mice (+/-) were treated with 1×10^14^ vg/kg mLB-001 at PND 1 (D1) or 5×10^13^ vg/kg mLB-001 at 8 weeks of age (W8). Terminal samples were analyzed at 6 months post-dosing. (A) Percentage of integrated albumin alleles in liver. (B) Fused mRNA expression in liver. (C) Circulating ALB-2A level. **** *P <* 0.0001, Student’s *t*-test.

To further characterize the mechanism of action of the GeneRide vector and to evaluate the correlation between genomic DNA integration and transgene mRNA and protein expression, we analyzed the 6-month post-dosing terminal liver samples from animals treated with mLB-001 at PND 1 (D1, 1×10^14^ vg/kg) or 8 weeks of age (W8, 5×10^13^ vg/kg). Site-specific genomic DNA integration in terminal liver tissues was determined using primers/probes designed to avoid detecting the episomal AAV genome [20]. All vehicle-treated animals showed undetectable levels of genomic DNA integration. Since mLB-001-edited hepatocytes in healthy heterozygous animals do not have a selective advantage, the percentage of the integrated alleles in these animals represented the baseline integration rate at the time of treatment. Regardless of the age at treatment, at 6 months post-dosing, we detected a greater than 100-fold increase in the percentage of integrated alleles in the MMA mice compared to the heterozygous mice (Fig 6A). These data suggest a significant expansion of the edited hepatocytes in the diseased animals, when treated as either neonates or adults. The fused *Alb-2A-Mmut* mRNA transcript expressed from the edited allele was quantified in liver tissues using a primer/probe set specific for the fused message. All vehicle-treated animals showed undetectable levels of fused mRNA. Following the same pattern of allelic integration analysis, at 6 months post-dosing with mLB-001, the expressed fused mRNA levels were greater than 100-fold higher in MMA mice than in the heterozygous animals, irrespective of the age at dosing (Fig 6B). Similarly, the ALB-2A fusion protein level in circulation was more than 100-fold higher in mLB-001-treated MMA mice than in the heterozygous mice when treated as either neonates or adults (Fig 6C). Thus, the direct correlation between transcription and translation from the edited allele in both heterozygous and MMA mice suggests that the increasing level of ALB-2A detected over time in the MMA mice was a direct result of cell expansion from the initially edited hepatocytes. MMUT holo-mutase activity in liver lysates was analyzed by enzymatic conversion of the substrate methylmalonyl-CoA to succinyl-CoA. Consistent with the lack of MMUT protein expression in the MMA mouse liver (Fig 3B), there was no mutase activity in the liver lysates of MMA mice treated with vehicle, while the mLB-001-treated MMA mice showed enzymatic activity comparable to or higher than the healthy heterozygous mice (S9 Fig). Immunoblot analysis of mitochondrial fractions of the liver tissues at 6 months post-dosing confirmed that the MMUT protein expressed from the transgene is correctly localized to mitochondria (S10 Fig).

At 6 months post-dosing, the percentage of MMUT-expressing hepatocytes in individual mLB-001-treated animals was variable. The cause of such variation might be multifactorial, including a mixed genetic background in this mouse line and variability in individual levels of food intake and protein metabolism, which led to different levels of hepatic stress driving varied expansion rates of the edited hepatocytes. Such variation allows correlation analyses among various molecular, biochemical and physiological readouts. The percentage of integrated alleles was linearly correlated with the fused mRNA and circulating ALB-2A levels (S11A Fig), and the percent of MMUT-expressing hepatocytes linearly correlated with the ALB-2A levels (S11B Fig). In addition, transgene expression, as represented by the surrogate biomarker ALB-2A in circulation, directly correlated with the animal body weight and anti-correlated with the disease biomarker, circulating methylmalonic acid levels (S11C Fig), suggesting that the expansion of the MMUT-expressing hepatocytes led to the improvement of the MMA disease phenotype.

### GeneRide vector successfully edited human hepatocytes in mice with a humanized liver

To translate the gene editing efficacy shown in the MMA mice to the human setting, we used the PXB mouse model, a chimeric mouse with more than 70% of the liver replaced with human hepatocytes [25]. The degree and durability of human hepatocyte engraftment was monitored by measuring plasma human albumin levels. The GeneRide vector used in this study, Vt196, was composed of the human hepatotropic capsid AAV-LK03 [26] encapsulating a vector genome containing the P2A sequence followed by the human *MMUT* coding sequence, flanked by two 1-kb homology guide sequences corresponding to the human *ALB* locus flanking the stop codon. The Vt196 design is the same as the vector used in the clinical studies, hLB-001 (NCT04581785, *ClinicalTrials*.*gov*) with the exception that the human *MMUT* coding sequence in hLB-001 is codon optimized while Vt196 contains the canonical sequence (GenBank Accession Number NM_000255.4). We intravenously injected adult PXB mice with 1×10^14^ or 5×10^13^ vg/kg of Vt196 and measured circulating ALB-2A. The animals were sacrificed at 8 weeks post-dosing, and liver tissue was analyzed for genomic DNA integration, fused *ALB-2A-MMUT* mRNA expression and MMUT protein expression by immunohistochemistry.

**Fig 7:**
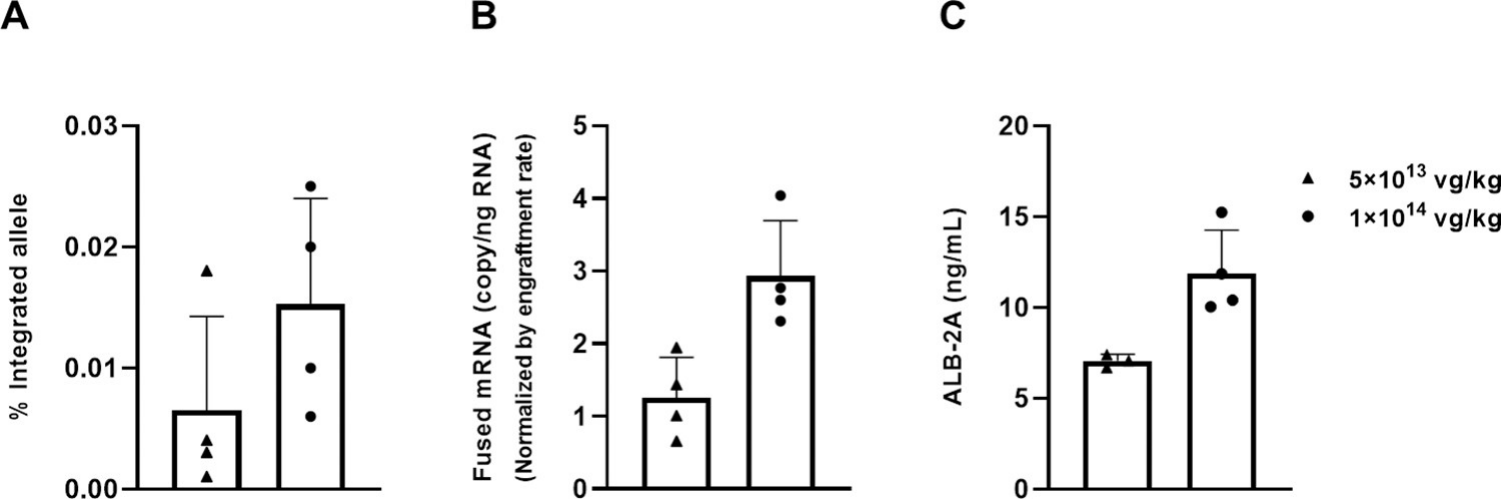
Analyses of terminal samples from PXB mice treated with Vt196. Adult PXB mice engrafted with human hepatocytes were treated with 1×10^14^ or 5×10^13^ vg/kg of Vt196 at 5 months of age. Animals were sacrificed at 8 weeks post-dosing and terminal samples were analyzed for (A) site-specific genomic DNA integration in the liver, (B) fused *ALB-2A-MMUT* mRNA expression in the liver, and (C) circulating ALB-2A concentration.

Because the study was performed in PXB mice with wild-type human hepatocytes, there was no selective advantage to drive the expansion of the edited hepatocytes. Therefore, the molecular analyses measured the basal level of genomic DNA integration resulting from homologous recombination, which took place in a dose-dependent manner in the humanized liver (Fig 7A), and resulted in dose-dependent production of fused *ALB-2A-MMUT* mRNA (Fig 7B) and fusion protein ALB-2A (Fig 7C). This study supports using a human hepatotropic capsid (LK03) and human homology sequences to deliver the vector genome into human hepatocytes to target site-specific integration into the *ALB* locus. Together, these results demonstrated that the GeneRide vector could edit human hepatocytes in vivo and express the therapeutic protein MMUT, supporting its use in treating MMA patients.

## Discussion

A strict protein-restricted diet is the mainstay management for methylmalonic acidemia. The principle is to reduce protein intake to limit the precursor amino acids for the dysfunctional propionic acid pathway while providing sufficient amino acids to support growth and avoid catabolic episodes [27]. In our mouse model of MMA, we provided a diet with reduced protein content (12%) compared to the standard rodent chow (21% protein) to mimic the clinical restriction of protein and to stabilize the severe and lethal phenotype observed in these mice. This facilitated the evaluation of the long-term therapeutic benefit of the GeneRide vector mLB-001, which we were not able to achieve with statistical significance due to the short life span of the animals fed with the standard chow [20].

We demonstrated that the low protein diet significantly improved MMA animal growth and survival and reduced circulating methylmalonic acid levels, the characteristic biomarker of the disease. However, this dietary protection was only transient, and animals gradually perished after returning to the standard chow. This mimicked clinical situations where MMA patients under strict dietary management continue to experience life-threatening metabolic decompensations triggered by events that induce catabolism such as infection, prolonged fasting, acute trauma, and excessive protein intake [21, 27], highlighting the need for better treatments to provide metabolic stability.

Under normal conditions, a healthy liver has a very low cell turnover, with <1% dividing hepatocytes as assessed by the expression of Ki67, a cell regeneration biomarker. However, the liver has a remarkable ability to regenerate following various types of injuries [28]. We found that under both low and standard protein diets, the liver of MMA mice had markedly elevated Ki67-positive hepatocytes, suggesting active tissue regeneration even when the disease was stabilized with the low protein diet. In MMA mice treated with mLB-001, the edited hepatocytes demonstrated a selective repopulation advantage, as measured by progressively increasing levels of the circulating fusion protein ALB-2A, the biomarker for corrected hepatocytes, regardless of the age of dosing and the dietary protein content.

During the first three months post-dosing under the low protein diet, vehicle- and mLB-001-treated MMA mice showed no significant difference in growth or survival. At 3 months post-dosing, to mimic the patient experience, metabolic stress was introduced by using a diet with higher protein content, increasing the metabolic flux through the propionate pathway. In response to this challenge, the MMA mice treated with mLB-001, either neonatally or as young adults, maintained their body weight, effectively controlled circulating methylmalonic acid levels, and demonstrated a clear improvement in animal health and survival compared to the vehicle-treated animals. The findings in this mouse model of severe MMA confirm a durable therapeutic effect of mLB-001 and support the prospect of a direct benefit in treating young MMA patients with a GeneRide vector. It should be noted that this mouse model does not recapitulate the neurological symptoms seen in MMA patients, therefore, we cannot be certain how effectively this technology may address the neurological sequelae of MMA patients.

MMA mice treated with mLB-001 expressed significant amounts of MMUT protein in the liver shortly after treatment. However, the expression was restricted to few hepatocytes, limiting the therapeutic effect of an intracellular protein even when expressed at a high level. As a result, mLB-001-treated MMA mice were still initially susceptible to metabolic decompensation. Control of the metabolic crisis requires expansion of corrected hepatocytes to populate a significant fraction of the liver. Interestingly, a recent report shows that using exogenous nucleases to increase the initial editing frequency can significantly reduce the treatment-to-efficacy time window [29].

GeneRide treatment did not normalize the animal growth or circulating methylmalonic acid levels during the time periods observed. This is consistent with the clinical observations that MMA patients receiving liver transplants showed improved, but not normalized, methylmalonic acid levels. These transplanted patients continue to require protein restriction, and may suffer neurological problems and die from metabolic strokes [30, 31], suggesting a limitation of liver-targeted therapies for this systemic disease.

Terminal sample analyses demonstrated that circulating ALB-2A levels positively correlated with an increase in the percent of edited hepatocytes, as measured by genomic DNA integration, fused mRNA expression and MMUT expression in the liver. In addition, the ALB-2A levels also positively correlated with physiological improvements in the MMA mice as demonstrated by improved body weight and reduced plasma methylmalonic acid levels. These results validate the utility of circulating ALB-2A as an effective GeneRide pharmacodynamic biomarker and a surrogate for both expansion of edited hepatocytes and MMUT protein expression, supporting its use in clinical trials to monitor real-time expansion of corrected hepatocytes.

It has recently been reported that AAV vectors randomly integrate at about 1–3% [32]. Importantly, the random integration appears to be ITR mediated and does not harness the power of homology directed repair, leading to rearrangements and deletions. We evaluated the random genome insertion of a GeneRide vector with human homology sequences in primary human hepatocytes. Using linear amplification-mediated PCR and deep sequencing to evaluate ITR-mediated rAAV integration [33, 34], we identified approximately 6,000 uniquely mappable insertion sites, with no signs for integration hotspots (unpublished results). Moreover, since the GeneRide vector does not include a promoter, the consequences of random integrations are expected to be benign.

Non-human primates have traditionally been used to evaluate gene therapy products as a preclinical translational tool, bridging mouse and human studies. However, for homology-guided gene editing, human targeted vectors with human homology guide sequences can only be effectively assessed in human cells. Humanized mouse models have emerged as a powerful tool for testing gene therapy agents [16, 35]. For homology-guided gene editing such as GeneRide, these mouse models offer a unique possibility to evaluate the activity of species-specific vectors. In this study, we utilized adult PXB mice that were highly repopulated with human hepatocytes and demonstrated that treatment with the human-specific GeneRide vector, Vt196, led to on-target genomic DNA integration, fused mRNA expression, MMUT and ALB-2A protein expression in the human hepatocytes. Compared to the levels obtained from the mouse studies using the same dosages of mLB-001, the Vt196-mediated genomic DNA integration rate was lower in PXB mice. Mechanisms underlying this reduction are yet to be elucidated though the following factors may be considered. Firstly, the vectors were composed of different capsids: AAV-DJ was used for the mouse-specific mLB-001 and AAV-LK03 was used for the human-specific Vt196. These capsids may have different biodistribution and pharmacokinetic properties in animals. Secondly, the transduction efficiency in hepatocytes of different species and origins might differ. For example, AAV-DJ can robustly transduce cells throughout the mouse liver [15], while AAV-LK03 preferentially transduces human hepatocytes in the periportal zone [16]. Thirdly, the PXB mice develop fatty liver and steatosis after transplantation [36], which may limit the tissue penetration and cell entry of the viral particles [37]. These factors may lead to a reduced gene editing rate in the human hepatocytes in the PXB mice. Nevertheless, the edited hepatocytes expressed the MMUT protein at high levels, as expected. The human hepatocytes were not MMUT deficient, therefore, there was no selective growth advantage of GeneRide-edited cells in the PXB mice. In a clinical setting, GeneRide editing is expected to restore the health of the diseased hepatocytes and support their expansion and liver repopulation through selective advantage. Such expansion could allow the edited hepatocytes to provide metabolic stability to MMA patients. In addition to evaluating safety, the ongoing Phase 1/2 clinical study of hLB-001 (NCT04581785, *ClinicalTrials*.*gov*) includes analyzing the surrogate biomarker ALB-2A to identify the presence and expansion of edited hepatocytes in pediatric MMA patients.

We have demonstrated in preclinical studies that GeneRide technology resulted in targeted genome editing and durable transgene expression in healthy and diseased hepatocytes. GeneRide technology utilizes a natural DNA repair process, homologous recombination, to enable precise editing of the genome and expression of transgenes without the need for exogenous nucleases or promoters that have been associated with an increased risk of immune response and cancer. On the other hand, the editing efficiency is low, and the number of edited cells may not be sufficient to produce therapeutic effects in diseases that require a high editing frequency to correct a high percentage of hepatocytes [38]. However, in preclinical models of diseases such as MMA that cause hepatic stress, the high liver turnover rate allows the corrected, healthy hepatocytes to repopulate the liver through a selective growth advantage, resulting in more corrected hepatocytes to provide therapeutic benefit. So far, we have demonstrated selective advantage of GeneRide vector-edited hepatocytes in three other preclinical disease models: alpha-1 antitrypsin deficiency [39], hereditary tyrosinemia type 1 and Wilson disease (unpublished results). Similar liver repopulation with healthy hepatocytes has been demonstrated using cell transplantation studies in other diseases such as progressive familial intrahepatic cholestasis [40]. These inborn errors of metabolism rank among the top indications for pediatric liver transplantation and could potentially be treated using the GeneRide technology described here. In addition, GeneRide technology could potentially be applied to treating other diseases that do not provide selective advantage by leveraging the high expression from the albumin promoter, including but not limited to diseases requiring modest levels of corrective protein expression for therapeutic benefit, such as Crigler-Najjar [41], or expression of secreted therapeutic proteins. Such opportunities potentially offer a one-dose, durable alternative to diseases currently managed with enzyme replacement therapy.

## Supporting information

S1 Fig Illustration of GeneRide technology and PCR designs. A GeneRide vector genome consists of species-specific homology guide sequences flanking the stop codon of the endogenous *ALB* gene, a peptide 2A and a coding sequence of methylmalonyl coenzyme A mutase. Homologous recombination between the vector genome and *ALB* locus results in the insertion of 2A-MMUT coding sequence in-frame with and upstream of the stop codon of the *ALB* gene. The transcription product is a fused *ALB-2A-MMUT* mRNA driven by the native *ALB* promoter. Upon translation, the 2A peptide mediates ribosomal skipping, resulting in two proteins, the secreted fusion protein ALB-2A and MMUT. At the genomic DNA level, the edited allele can be quantified using long-range PCR amplification with primers F1 and R1, followed by a qPCR assay using primers F1 and R2 with probe P1. The fused mRNA can be quantified by ddPCR using primers F2 and R3 with probe P2.
(TIF)

S2 Fig Circulating alanine aminotransferase activity in 4-week-old mice. Heterozygous (*Mmut*^*+/-*^) and MMA (*Mmut*^*-/-*^) mice were fed standard chow (21% protein content) and plasma samples from 4-week-old mice were measured for alanine aminotransferase activity. ** *P* < 0.01, Student’s *t*-test.
(TIF)

S3 Fig Immunohistochemistry analysis of Ki67 in 10-week-old mice. Heterozygous (*Mmut*^*+/-*^) and MMA (*Mmut*^*-/-*^) mice were fed standard chow (21% protein content). Animals were killed at 7–10 weeks of age, and liver tissues were subjected to immunohistochemistry analysis for Ki67. Ten 0.4 mm^2^ regions were randomly selected from each tissue section and Ki67-positive cells were counted by staff blinded to the sample identity. A representative sample of each is shown. * *P* < 0.05, Student’s *t*-test. The scale bars represent 100 μm.
(TIF)

S4 Fig Kaplan-Meier survival curve of MMA mice fed with the standard rodent chow containing 21% protein. MMA mice were fed standard chow (21% protein content). Among the 30 animals, 25 were found dead, 3 were euthanized due to moribund state (prostration, decreased motor activity, inability to right, cold to touch, pale, and/or tremors), and 2 were terminated at 7.5 months of age at the end of the study.
(TIF)

S5 Fig Kaplan-Meier survival curves of MMA mice on a transient low protein diet. Nursing dams were on a 21% protein diet (standard chow) until pups were PND 14. Randomly selected litters were provided with a 12% protein diet between PND 14 and 49 and then returned to the 21% protein diet. The rest of the litters were on a 21% protein diet throughout their life span. Color-matched arrows on top represent the time periods in which the % protein diets were provided. All pups were weaned on PND 28. Log-rank test demonstrated a significantly better survival of MMA mice provided with the 12% protein diet (*P <* 0.01), indicating that a transient, low protein diet can offer significant benefit to the MMA mice, although the effect is only temporary.
(TIF)

S6 Fig Body weight of MMA mice and their heterozygous littermates. (A) Animals were dosed with vehicle or 1×10^14^ vg/kg mLB-001 on PND 1. Nursing dams were kept on the standard 21% protein diet until PND 14, when the diet was switched to a 12% protein diet. Pups were weaned on PND 28 and maintained on the 12% protein diet. At 3 months of age, all surviving animals were switched to the standard chow with 21% protein content and then to a 40% protein diet at 4 months of age. Animals were monitored until 6 months of age. (B) Animals were dosed with vehicle, 2.5×10^13^, 5×10^13^ or 1×10^14^ vg/kg mLB-001 on PND 1. Other procedures are the same as for (A). (C) Animals were maintained on the 12% protein diet and dosed with vehicle or 5×10^13^ vg/kg mLB-001 at 8 weeks of age. At 3 months post-dosing, the diet was switched to the standard chow with 21% protein content and then to a 40% protein diet at 4 months post-dosing. Animals were monitored until 8 months of age.
(TIF)

S7 Fig Serum albumin concentrations of MMA mice treated with vehicle or mLB-001 on PND 1. Animals were injected with vehicle or mLB-001 at PND1. Blood samples were collected monthly post-dosing and subjected to ELISA for mouse total albumin. Two-way analysis of variance reveals no significant difference among the dosing groups and no significant change over time for all groups.
(TIF)

S8 Fig Immunohistochemistry analysis of MMUT in the liver of an MMA mouse treated with mLB-001 at 8 weeks of age. Animals were injected with 5×10^13^ vg/kg mLB-001 at 8 weeks of age. Terminal liver tissues were collected at 6 months post-dosing and subjected to immunohistochemistry analysis for MMUT. Images of a representative animal is shown. The scale bars represent 1 mm (left) and 200 μm (right).
(TIF)

S9 Fig MMUT holo-mutase activity in liver. MMA mice (-/-) or heterozygous mice (+/-) were treated with 1×10^14^ vg/kg mLB-001 at PND 1 (D1) or 5×10^13^ vg/kg mLB-001 at 8 weeks of age (W8). Terminal samples were analyzed at 6 months post-dosing. The mutase activity unit is defined as the amount of the enzyme that converted methylmalonyl CoA to succinyl CoA (μmol/min), and the data were normalized by the total protein in the liver lysates.
(TIF)

S10 Fig Immunoblot analysis of MMUT in the liver mitochondrial fraction of mice treated with mLB-001 as neonates or adults. Animals were injected with vehicle or mLB-001 on PND 1 (A, Neonates) or at 8 weeks of age (B, Adults). Terminal liver tissues were collected at 6 months post-dosing and mitochondrial fractions were isolated. An MMA mouse and a heterozygote (Het) treated with vehicle were used as controls. The full-length MMUT protein is expected to be ~80 KD. The ~50 KD bands are MMUT-specific and were derived from both endogenous and mLB-001 delivered proteins. Mitochondrial protein cytochrome C was used as a loading control.
(TIF)

S11 Fig Correlations of terminal parameters in MMA mice treated with mLB-001 on PND 1. Animals were killed at 6 months of age and terminal samples were analyzed for liver genomic DNA integration, liver fused mRNA level, liver MMUT expression by immunohistochemistry, plasma ALB-2A by ELISA and methylmalonic acid by LC-MS/MS. Straight lines represent the best fit to the data by simple linear regression. In these mice, 1000 μg/mL ALB-2A was equivalent to 5% of total albumin. * *P <* 0.05, ** *P <* 0.01, *** P < 0.001, **** *P <* 0.0001, F-test for non-0 slope hypothesis.
(TIF)

S1 Movie Activities of MMA mice injected with vehicle or mLB-001. Animals were injected with vehicle or 5×10^13^ vg/kg mLB-001 at 8 weeks of age. At 5 months post-dosing, two animals, one from each treatment group, were removed from their home cages and placed together in a freshly prepared cage. The video was recorded within 2 minutes of the animal placement in the cage.
(GIF)

S1 Raw images Original blot images. (PDF)

S1 Table Raw data used to generate figures. (PDF)
