# Supplementary figures and images for: Novel AAV-mediated genome editing therapy improves health and survival in a mouse model of methylmalonic acidemia
Source: PLoS One. 2022 Sep 20;17(9):e0274774. doi: 10.1371/journal.pone.0274774 (PMC9488783; doi:10.1371/journal.pone.0274774)

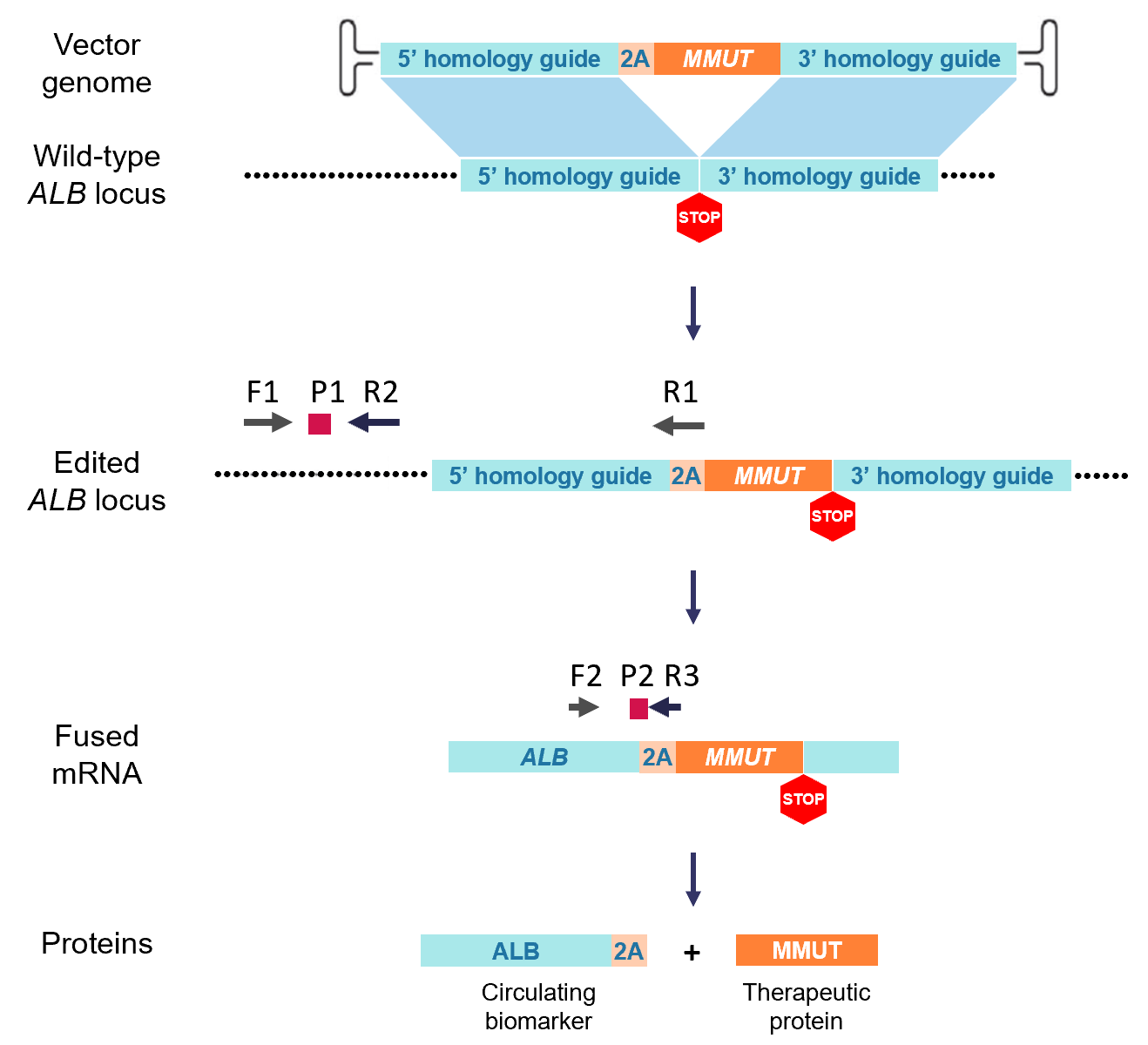

Supplement: S1 Fig — A GeneRide vector genome consists of species-specific homology guide sequences flanking the stop codon of the endogenous ALB gene, a peptide 2A and a coding sequence of methylmalonyl coenzyme A mutase. Homologous recombination between the vector genome and ALB locus results in the insertion of 2A-MMUT coding sequence in-frame with and upstream of the stop codon of the ALB gene. The transcription product is a fused ALB-2A-MMUT mRNA driven by the native ALB promoter. Upon translation, the 2A peptide mediates ribosomal skipping, resulting in two proteins, the secreted fusion protein ALB-2A and MMUT. At the genomic DNA level, the edited allele can be quantified using long-range PCR amplification with primers F1 and R1, followed by a qPCR assay using primers F1 and R2 with probe P1. The fused mRNA can be quantified by ddPCR using primers F2 and R3 with probe P2. (TIF) [file pone.0274774.s001.tif]

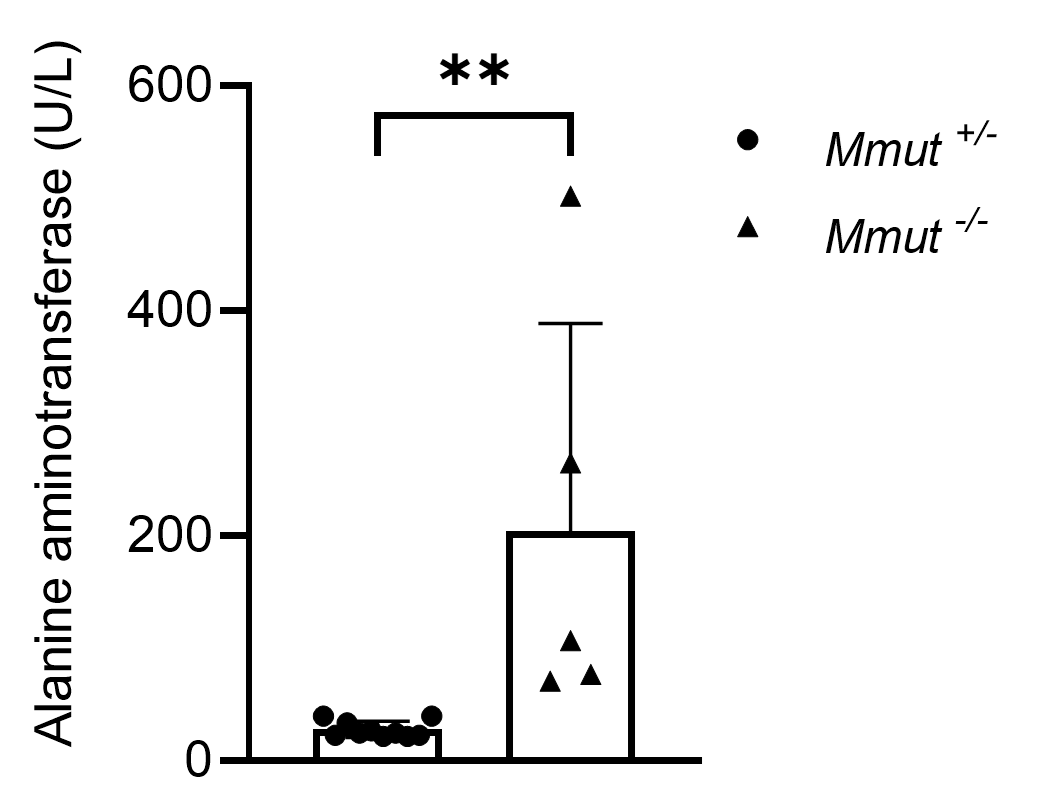

Supplement: S2 Fig — Heterozygous (Mmut+/-) and MMA (Mmut-/-) mice were fed standard chow (21% protein content) and plasma samples from 4-week-old mice were measured for alanine aminotransferase activity. ** P < 0.01, Student’s t-test. (TIF) [file pone.0274774.s002.tif]

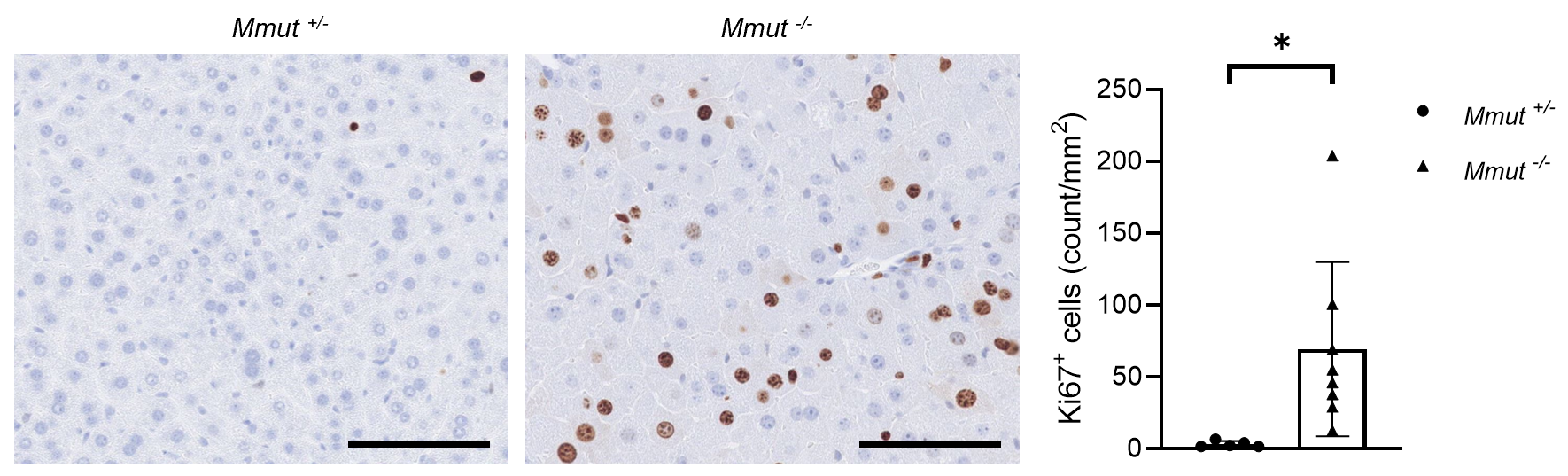

Supplement: S3 Fig — Heterozygous (Mmut+/-) and MMA (Mmut-/-) mice were fed standard chow (21% protein content). Animals were killed at 7–10 weeks of age, and liver tissues were subjected to immunohistochemistry analysis for Ki67. Ten 0.4 mm2 regions were randomly selected from each tissue section and Ki67-positive cells were counted by staff blinded to the sample identity. A representative sample of each is shown. * P < 0.05, Student’s t-test. The scale bars represent 100 μm. (TIF) [file pone.0274774.s003.tif]

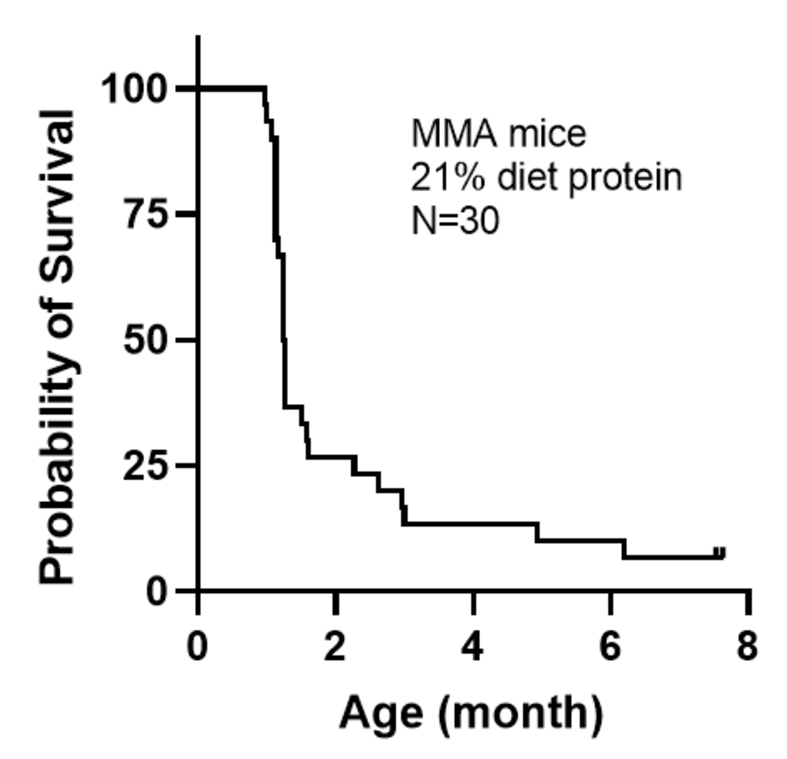

Supplement: S4 Fig — MMA mice were fed standard chow (21% protein content). Among the 30 animals, 25 were found dead, 3 were euthanized due to moribund state (prostration, decreased motor activity, inability to right, cold to touch, pale, and/or tremors), and 2 were terminated at 7.5 months of age at the end of the study. (TIF) [file pone.0274774.s004.tif]

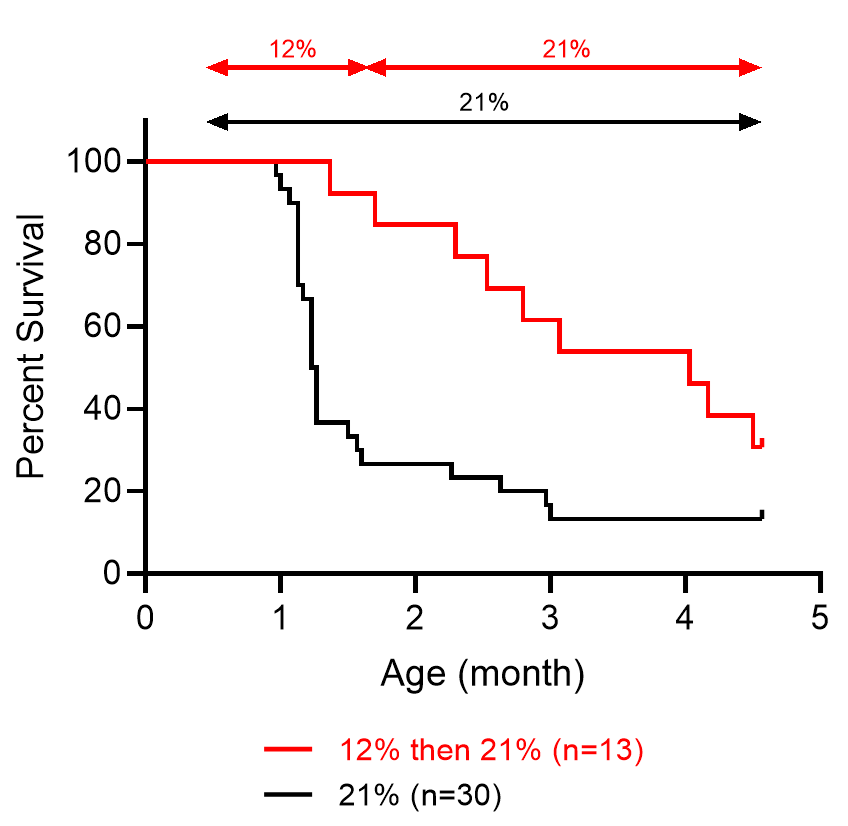

Supplement: S5 Fig — Nursing dams were on a 21% protein diet (standard chow) until pups were PND 14. Randomly selected litters were provided with a 12% protein diet between PND 14 and 49 and then returned to the 21% protein diet. The rest of the litters were on a 21% protein diet throughout their life span. Color-matched arrows on top represent the time periods in which the % protein diets were provided. All pups were weaned on PND 28. Log-rank test demonstrated a significantly better survival of MMA mice provided with the 12% protein diet (P < 0.01), indicating that a transient, low protein diet can offer significant benefit to the MMA mice, although the effect is only temporary. (TIF) [file pone.0274774.s005.tif]

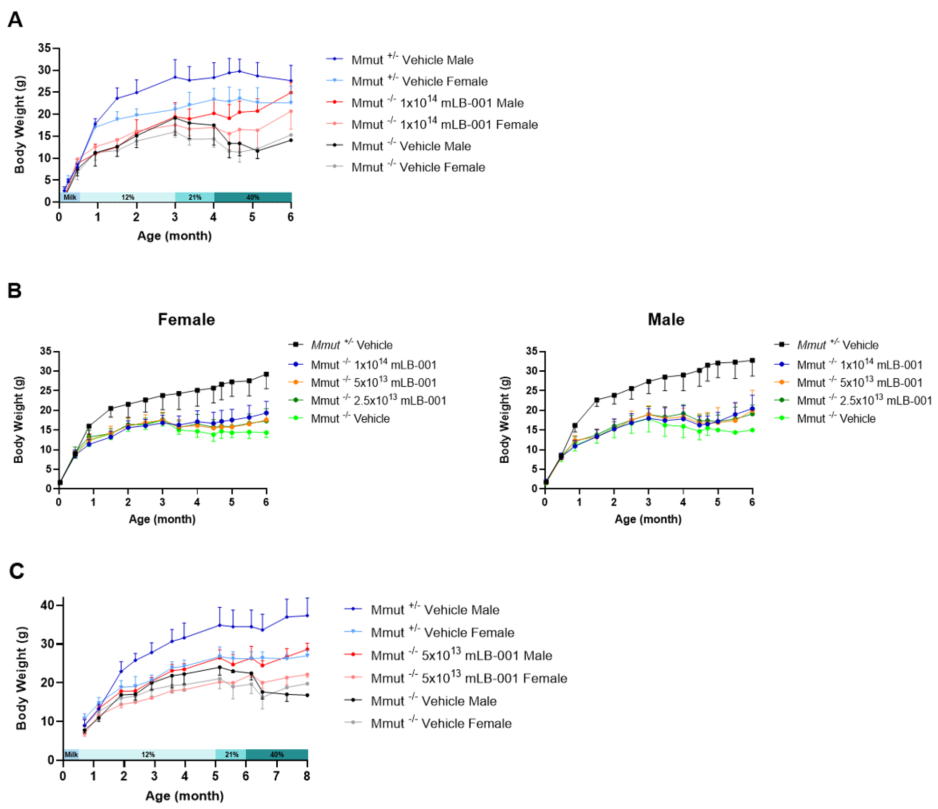

Supplement: S6 Fig — (A) Animals were dosed with vehicle or 1×10^14^ vg/kg mLB-001 on PND 1. Nursing dams were kept on the standard 21% protein diet until PND 14, when the diet was switched to a 12% protein diet. Pups were weaned on PND 28 and maintained on the 12% protein diet. At 3 months of age, all surviving animals were switched to the standard chow with 21% protein content and then to a 40% protein diet at 4 months of age. Animals were monitored until 6 months of age. (B) Animals were dosed with vehicle, 2.5×10^13^, 5×10^13^ or 1×10^14^ vg/kg mLB-001 on PND 1. Other procedures are the same as for (A). (C) Animals were maintained on the 12% protein diet and dosed with vehicle or 5×10^13^ vg/kg mLB-001 at 8 weeks of age. At 3 months post-dosing, the diet was switched to the standard chow with 21% protein content and then to a 40% protein diet at 4 months post-dosing. Animals were monitored until 8 months of age. (TIF) [file pone.0274774.s006.tif]

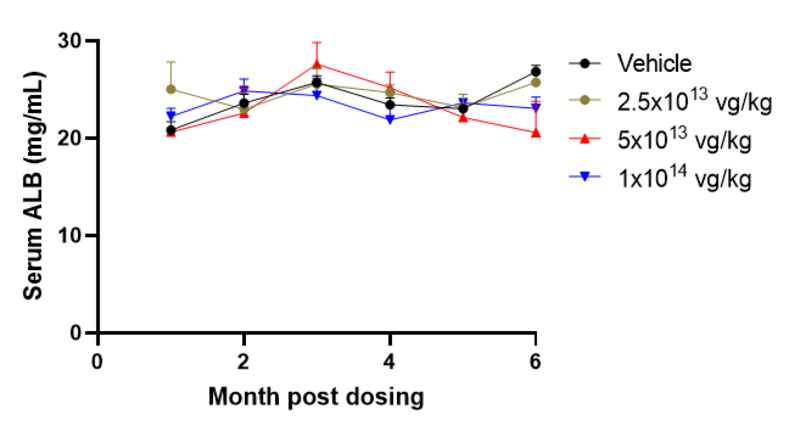

Supplement: S7 Fig — Animals were injected with vehicle or mLB-001 at PND1. Blood samples were collected monthly post-dosing and subjected to ELISA for mouse total albumin. Two-way analysis of variance reveals no significant difference among the dosing groups and no significant change over time for all groups. (TIF) [file pone.0274774.s007.tif]

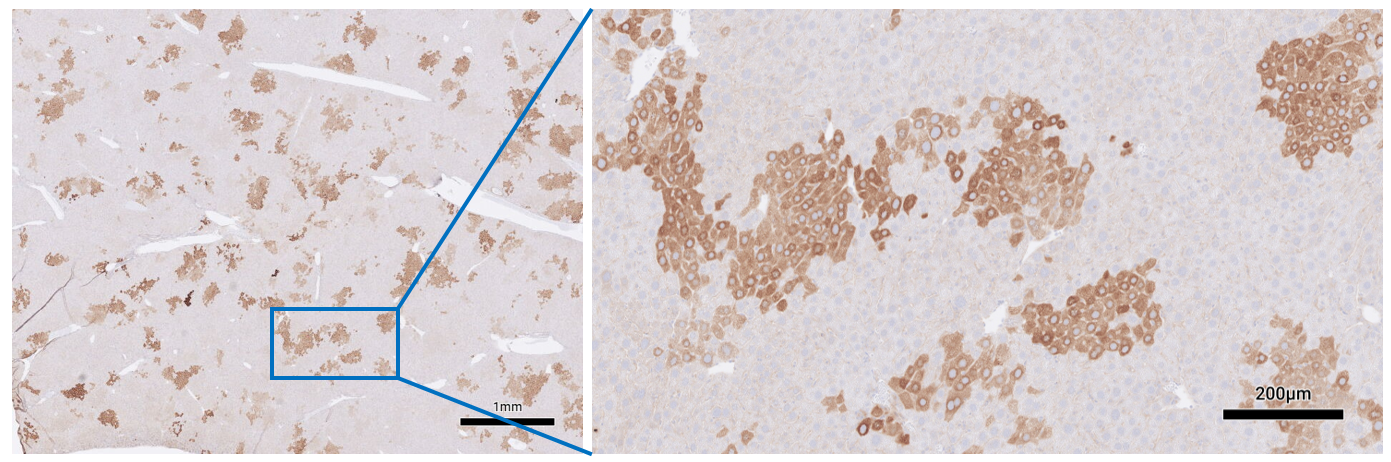

Supplement: S8 Fig — Animals were injected with 5×10^13^ vg/kg mLB-001 at 8 weeks of age. Terminal liver tissues were collected at 6 months post-dosing and subjected to immunohistochemistry analysis for MMUT. Images of a representative animal is shown. The scale bars represent 1 mm (left) and 200 μm (right). (TIF) [file pone.0274774.s008.tif]

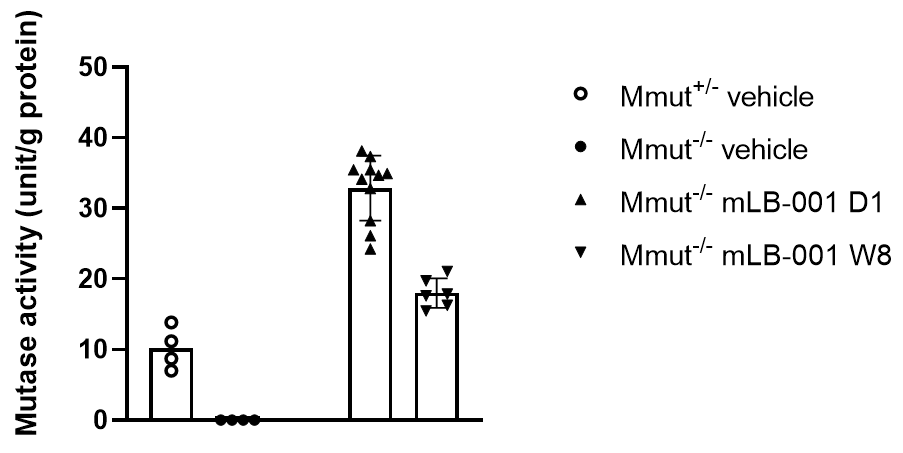

Supplement: S9 Fig — MMA mice (-/-) or heterozygous mice (+/-) were treated with 1×10^14^ vg/kg mLB-001 at PND 1 (D1) or 5×10^13^ vg/kg mLB-001 at 8 weeks of age (W8). Terminal samples were analyzed at 6 months post-dosing. The mutase activity unit is defined as the amount of the enzyme that converted methylmalonyl CoA to succinyl CoA (μmol/min), and the data were normalized by the total protein in the liver lysates. (TIF) [file pone.0274774.s009.tif]

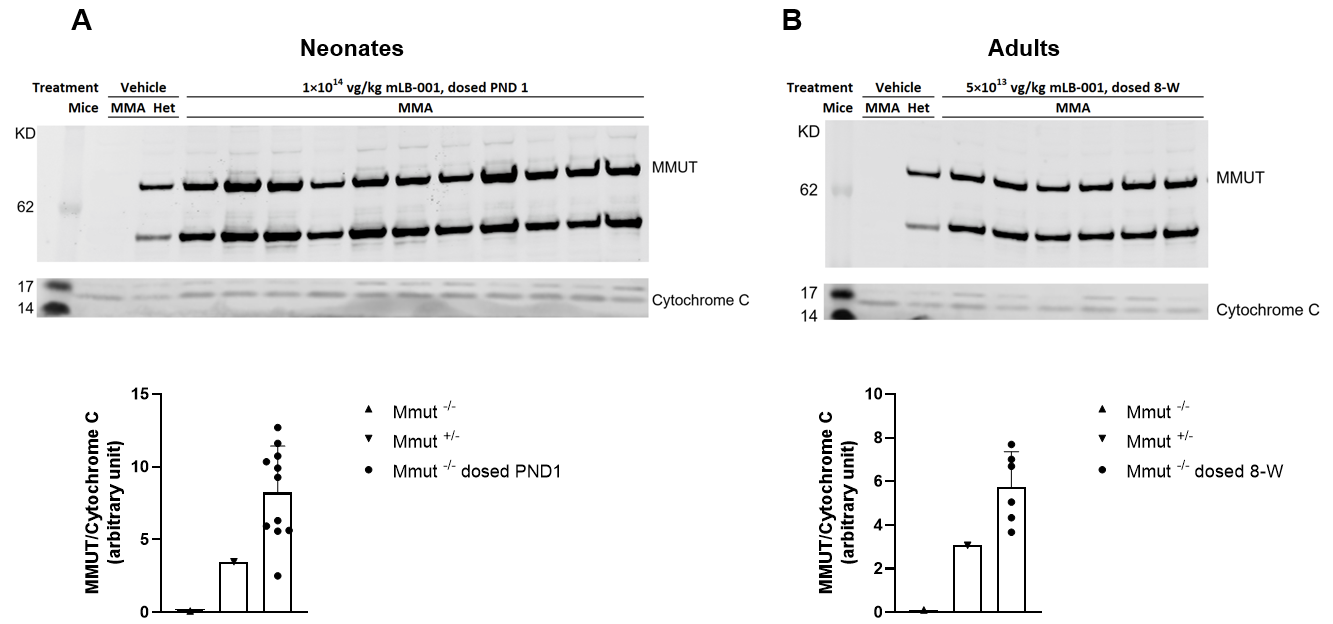

Supplement: S10 Fig — Animals were injected with vehicle or mLB-001 on PND 1 (A, Neonates) or at 8 weeks of age (B, Adults). Terminal liver tissues were collected at 6 months post-dosing and mitochondrial fractions were isolated. An MMA mouse and a heterozygote (Het) treated with vehicle were used as controls. The full-length MMUT protein is expected to be ~80 KD. The ~50 KD bands are MMUT-specific and were derived from both endogenous and mLB-001 delivered proteins. Mitochondrial protein cytochrome C was used as a loading control. (TIF) [file pone.0274774.s010.tif]

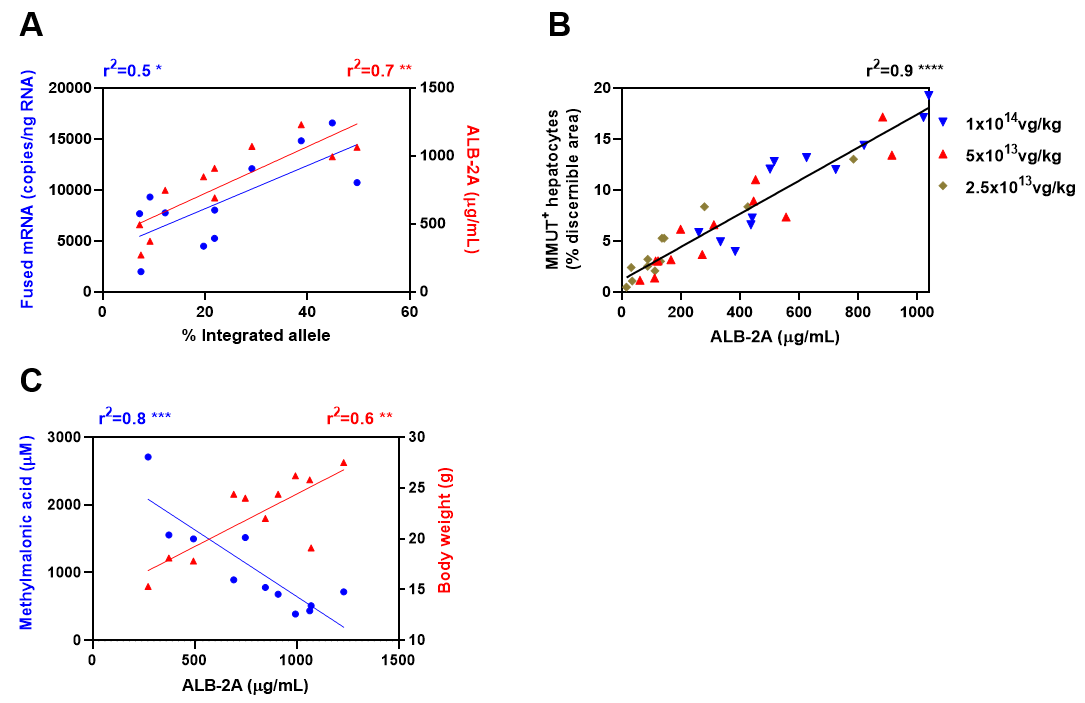

Supplement: S11 Fig — Animals were killed at 6 months of age and terminal samples were analyzed for liver genomic DNA integration, liver fused mRNA level, liver MMUT expression by immunohistochemistry, plasma ALB-2A by ELISA and methylmalonic acid by LC-MS/MS. Straight lines represent the best fit to the data by simple linear regression. In these mice, 1000 μg/mL ALB-2A was equivalent to 5% of total albumin. * P < 0.05, ** P < 0.01, *** P < 0.001, **** P < 0.0001, F-test for non-0 slope hypothesis. (TIF) [file pone.0274774.s011.tif]

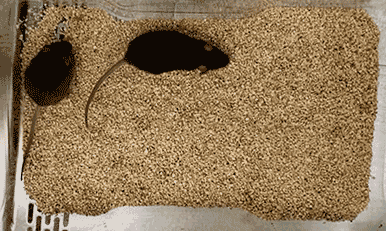

Supplement: S1 Movie — Animals were injected with vehicle or 5×10^13^ vg/kg mLB-001 at 8 weeks of age. At 5 months post-dosing, two animals, one from each treatment group, were removed from their home cages and placed together in a freshly prepared cage. The video was recorded within 2 minutes of the animal placement in the cage. (GIF) [file pone.0274774.s012.gif]
