# Supplementary material for: Novel AAV-mediated genome editing therapy improves health and survival in a mouse model of methylmalonic acidemia
Source: PLoS One. 2022 Sep 20;17(9):e0274774. doi: 10.1371/journal.pone.0274774 (PMC9488783; doi:10.1371/journal.pone.0274774)
Supplement: S1 Raw images — (PDF) [file pone.0274774.s013.pdf]

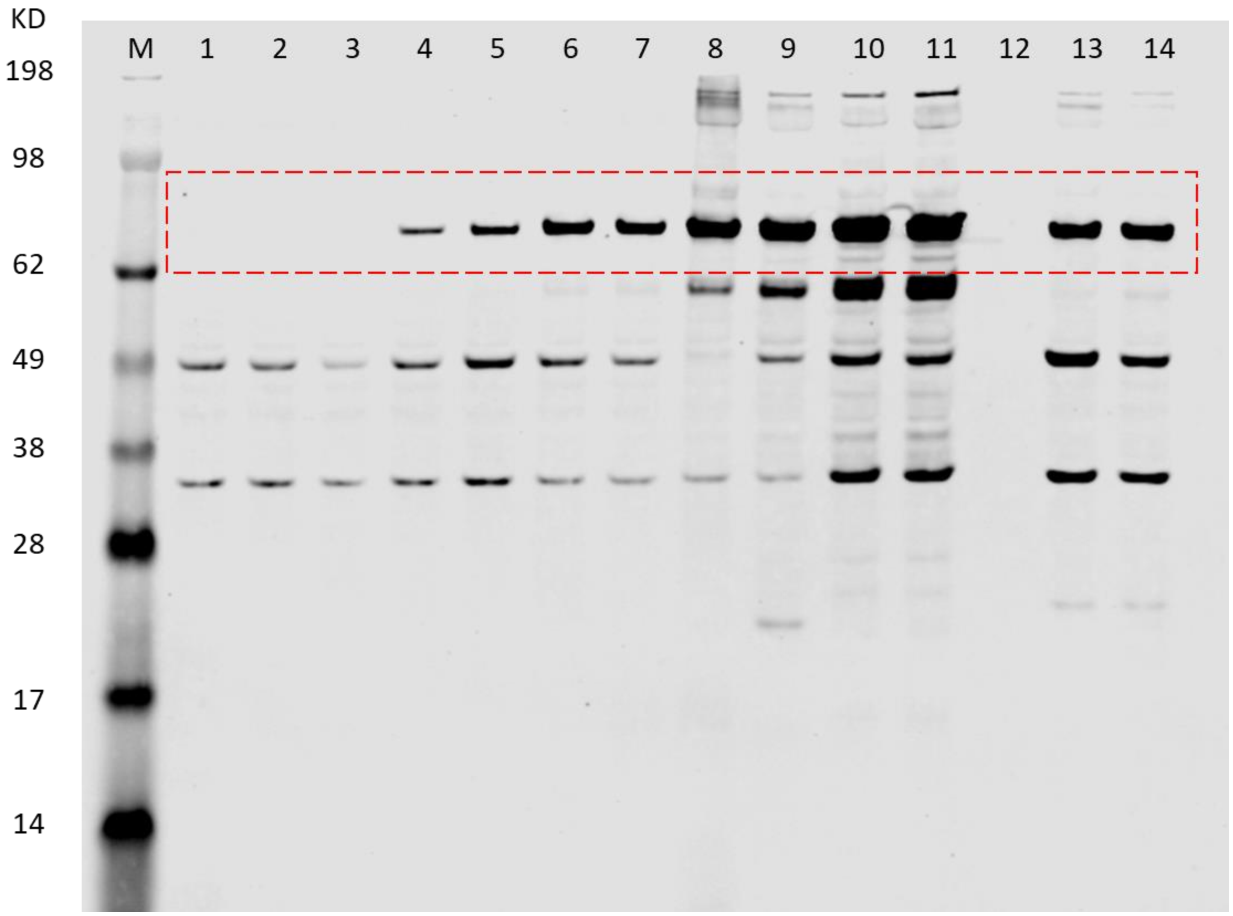

S1 Blot. Original blot image for Fig 3B, MMUT.

Loading order:
M: Molecular weight ladder (apparent weights (KD) are indicated on the left)
1-3: Three MMA mice at 1-month old
4-7: Four MMA mice treated with mLB-001 at PND1, 1-month post dosing
8: An MMA mouse treated with mLB-001 at PND1, 2-months post dosing
9: An MMA mouse treated with mLB-001 at PND1, 3-months post dosing
10-11: Two MMA mice treated with mLB-001 at PND1, 7-months post dosing
12: Blank
13-14: Two heterozygous mice at 2-months old

Image was captured with an Odyssey DLx Imager.

Fig 3B upper panel image was generated from the area in the red frame.

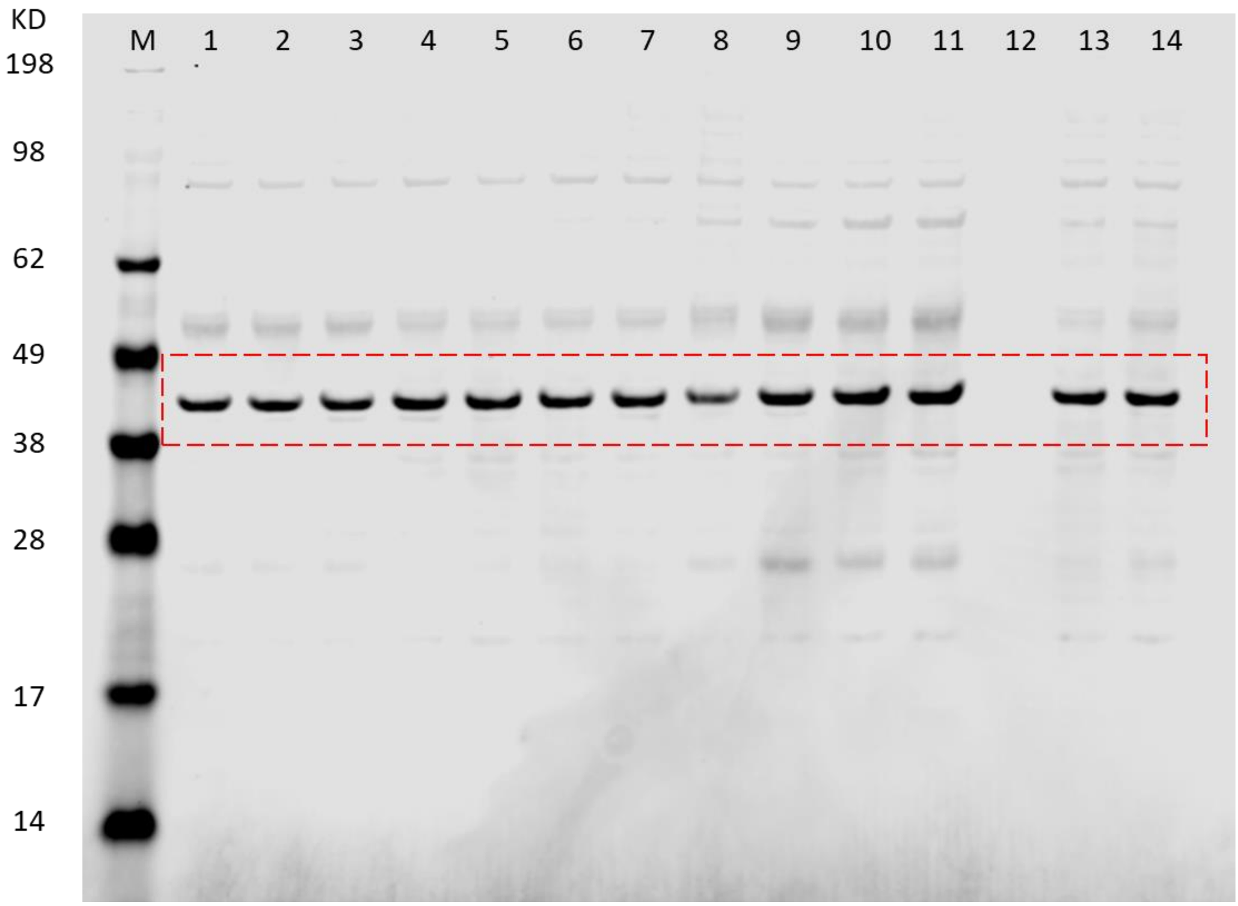

S2 Blot. Original blot image for Fig 3B, actin.

Loading order:
M: Molecular weight ladder (apparent weights (KD) are indicated on the left)
1-3: Three MMA mice at 1-month old
4-7: Four MMA mice treated with mLB-001 at PND1, 1-month post dosing
8: An MMA mouse treated with mLB-001 at PND1, 2-months post dosing
9: An MMA mouse treated with mLB-001 at PND1, 3-months post dosing
10-11: Two MMA mice treated with mLB-001 at PND1, 7-months post dosing
12: Blank
13-14: Two heterozygous mice at 2-months old

Image was captured with an Odyssey DLx Imager.

Fig 3B lower panel image was generated from the area in the red frame.

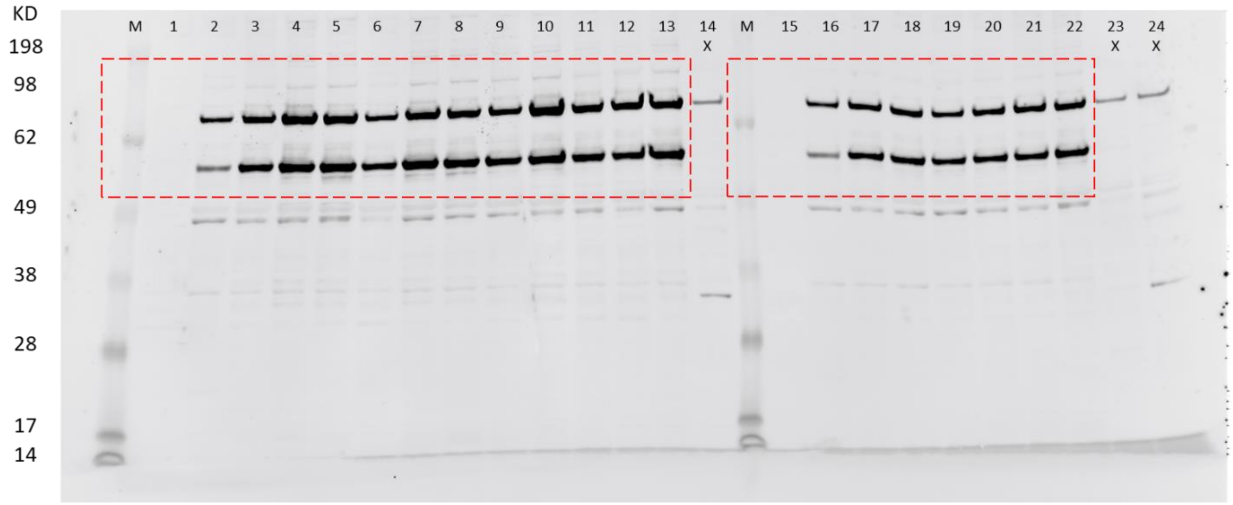

S3 Blot. Original blot image for S10 Fig, MMUT.

Loading order:
M: Molecular weight ladder (apparent weights (KD) are indicated on the left)
1: A vehicle-treated MMA mouse at 6-months old
2: A vehicle-treated heterozygous mouse at 6-months old
3-13: Eleven MMA mice treated with mLB-001 at PND1, 6-months post dosing
14: A heterozygous mouse at 6-months old, whole cell lysate (not shown)
M: Molecular weight ladder
15: A vehicle-treated MMA mouse at 8-months old
16: A vehicle-treated heterozygous mouse at 8-months old
17-22: Six MMA mice treated with mLB-001 at 8-weeks old, 6-months post dosing
23-24: Two heterozygous mice at 8-months old, whole cell lysates (not shown)

Image was captured with an Odyssey DLx Imager.

S10 Fig A upper panel image was generated from the area in the red frame on the left.
S10 Fig B upper panel image was generated from the area in the red frame on the right.

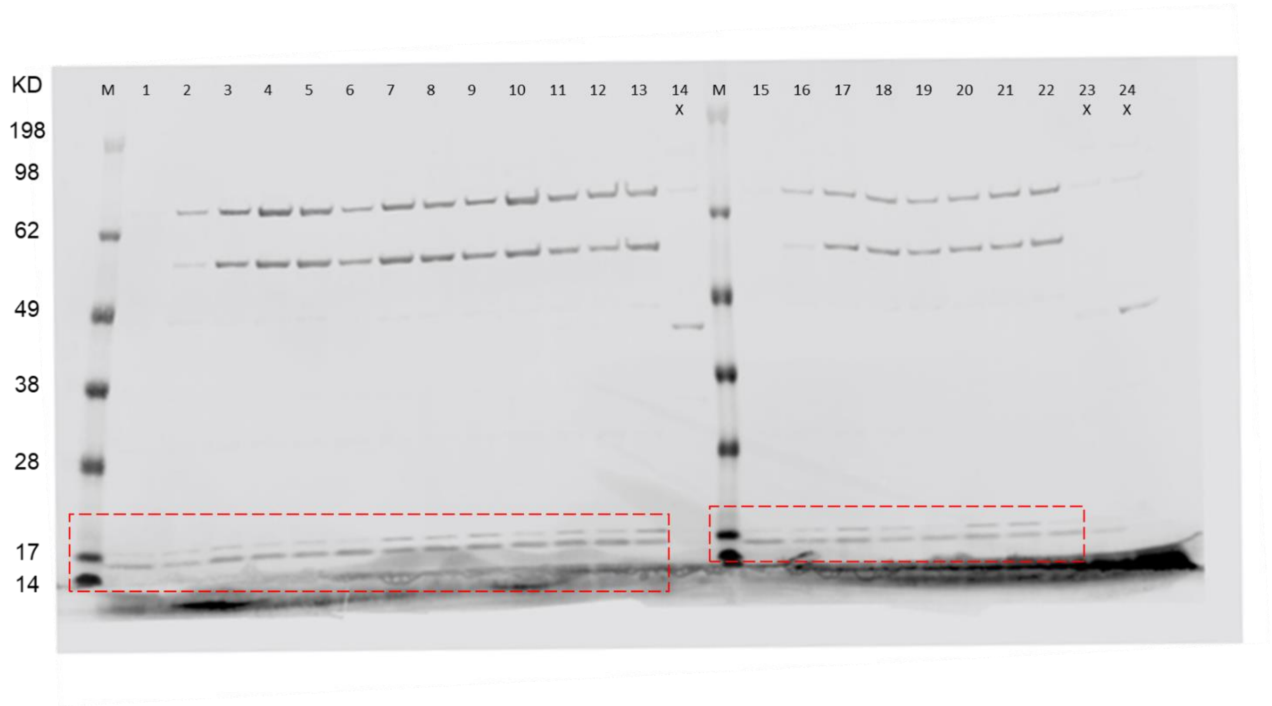

S4 Blot. Original blot image for S10 Fig, cytochrome c.

Loading order:
M: Molecular weight ladder (apparent weights (KD) are indicated on the left)
1: A vehicle-treated MMA mouse at 6-months old
2: A vehicle-treated heterozygous mouse at 6-months old
3-13: Eleven MMA mice treated with mLB-001 at PND1, 6-months post dosing
14: A heterozygous mouse at 6-months old, whole cell lysate (not shown)
M: Molecular weight ladder
15: A vehicle-treated MMA mouse at 8-months old
16: A vehicle-treated heterozygous mouse at 8-months old
17-22: Six MMA mice treated with mLB-001 at 8-weeks old, 6-months post dosing
23-24: Two heterozygous mice at 8-months old, whole cell lysates (not shown)

Image was captured with an Odyssey DLx Imager.

S10 Fig A lower panel image was generated from the area in the red frame on the left.
S10 Fig B lower panel image was generated from the area in the red frame on the right.
