# Supplementary material for: Novel AAV-mediated genome editing therapy improves health and survival in a mouse model of methylmalonic acidemia
Source: PLoS One. 2022 Sep 20;17(9):e0274774. doi: 10.1371/journal.pone.0274774 (PMC9488783; doi:10.1371/journal.pone.0274774)
Supplement: S1 Table — (PDF) [file pone.0274774.s014.pdf]

S1 Table. Raw data set for Fig. 2C. Change in body weight (%) of MMA mice treated with vehicle or 1×10^{14} vg/kg mLB-001 on PND 1

Animal	Treatment	Age (month)												
		**0.5**	**0.7**	**0.9**	**1.5**	**1.7**	**1.9**	**3.0**	**3.4**	**4.0**	**4.4**	**4.7**	**5.1**	**6.0**
V1	Vehicle	40.7	51.8	62.1	71.0	73.8	90.3	100.0	92.4	88.3	69.7	68.3	73.8	
V2	Vehicle	37.4	50.2	76.8	76.1	78.1	91.0	100.0	84.5	85.2	69.7	66.5		
V3	Vehicle	36.1	44.6	68.0	67.5	68.6	79.9	100.0	87.6	85.8	71.6			
V4	Vehicle	53.2	62.7	61.4	78.4	84.2	87.1	100.0	93.6	98.8	78.4	81.9	79.5	89.5
V5	Vehicle			59.3	62.5	67.3	76.6	100.0	84.7					
V6	Vehicle			63.0	59.7	65.4	75.4	100.0	94.3	99.5	79.1	75.8		
V7	Vehicle	38.9	49.5	64.4	63.8	73.2	83.2	100.0	86.6	83.2	68.5	69.8	69.8	
V8	Vehicle	44.5	54.5	70.3	65.9	73.1	69.8	100.0						
V9	Vehicle	51.2	57.4	63.7	81.0	85.1	92.9	100.0	108.3	113.7	78.6	81.5	76.8	83.9
T1	1e14 vg/kg			76.4	77.5	84.3	91.6	100.0	96.6	101.1	79.8	80.9	78.1	101.7
T2	1e14 vg/kg			81.3	96.3	100.6	102.5	100.0	116.3	101.3	81.3	93.1	83.1	111.3
T3	1e14 vg/kg	50.0	56.4	63.4	71.8	76.7	86.6	100.0	88.1	98.0	101.0	110.4	111.4	129.7
T4	1e14 vg/kg	55.5	61.8	65.4	81.7	86.9	89.5	100.0	84.8	100.5	101.0	108.9	109.4	127.7
T5	1e14 vg/kg	48.9	60.7	78.2	80.9	81.4	91.5	100.0	93.6	96.3	96.8	105.9	102.1	127.7
T6	1e14 vg/kg	60.5	80.3	80.9	80.9	80.9	94.7	100.0	99.3	90.8	81.6	73.7	78.3	100.7
T7	1e14 vg/kg			57.5	76.9	86.3	91.3	100.0	88.8	86.3	72.5	76.3	79.4	119.4
T8	1e14 vg/kg			57.1	65.1	71.8	83.6	100.0	91.6	92.4	76.9	80.7	86.1	92.4
T9	1e14 vg/kg	51.8	61.9	69.4	67.4	72.5	81.3	100.0	102.1	120.2	122.8	123.8	124.9	133.7
T10	1e14 vg/kg	47.5	51.7	63.4	65.6	73.2	78.7	100.0	95.6	106.0	95.6	112.6	114.2	150.3
T11	1e14 vg/kg		38.8	60.5	69.8	73.5	80.2	100.0	103.7	100.0	104.3	111.7	107.4	150.6

S2 Table. Raw data set for Fig. 2D. Circulating methylmalonic acid levels (µM) in MMA mice treated with vehicle or 1×10^{14} vg/kg mLB-001 on PND 1

**Animal**	**Treatment**	**Age (month)**					
		**1**	**2**	**3**	**4**	**4.7**	**6**
V1	Vehicle	996	651	1104	2124	1179	
V2	Vehicle	1136	746	965	795	2848	
V3	Vehicle	776	547	435	1242	1823	
V4	Vehicle	855	689	552	1202	975	1245
V5	Vehicle	847	506	453			
V6	Vehicle	876	421	697	1044		
V7	Vehicle	930	509	462	501	921	
V8	Vehicle	616	590	556			
V9	Vehicle	1027	677	541	717	1716	2229
V10	Vehicle	1216					
T1	$1x10^{14}$ vg/kg	972		666	636	550	680
T2	$1x10^{14}$ vg/kg	869	715	589	522	525	388
T3	$1x10^{14}$ vg/kg	1189	452	444	1954	981	1518
T4	$1x10^{14}$ vg/kg	843	488	661	1050	1719	2708
T5	$1x10^{14}$ vg/kg	1466	741	443	1163	807	436
T6	$1x10^{14}$ vg/kg	1821	720	300	429	346	716
T7	$1x10^{14}$ vg/kg	1248	1471	725	1440	1235	893
T8	$1x10^{14}$ vg/kg	1079	615	476	887	2162	510
T9	$1x10^{14}$ vg/kg	594	448	469	685	756	1555
T10	$1x10^{14}$ vg/kg	510	541	564	688	1144	1498
T11	$1x10^{14}$ vg/kg	793	549	405	531	750	780
T12	$1x10^{14}$ vg/kg	3310					
T13	$1x10^{14}$ vg/kg	655					

S3 Table. Raw data set for Fig. 3A. Plasma ALB-2A levels (μg/mL) in heterozygous and MMA mice treated with 1×10^{14} vg/kg mLB-001 on PND 1

Animal	Age (month)				
	**1**	**2**	**3**	**4**	**6**
MMA 1		23.9	72.8	507.6	994.5
MMA 2	2.1	9.2	18.6	68.3	272.1
MMA 3	2.5	12.3	13.7	95.4	373.6
MMA 4	4.5	17.6	32.5	115.8	494.4
MMA 5	2.6	17.5	40.3	344.6	691.8
MMA 6	2.0	15.9	29.5	153.5	748.6
MMA 7	3.2	22.9	50.1	304.2	847.1
MMA 8	2.1		68.7	319.5	909.8
MMA 9	3.0	41.1	53.8	435.9	1064.7
MMA 10	3.7	34.3	85.8	327.9	1070.9
MMA 11	2.8	20.0	145.3	850.0	1231.1
Heterozygous 1	1.4	1.6	1.9	2.1	1.5
Heterozygous 2	1.8	1.7	1.8	2.4	2.7
Heterozygous 3	1.8	2.0	2.4	3.0	3.3
Heterozygous 4	1.8	2.0	2.1	2.2	
Heterozygous 5	1.6	1.8	2.4	2.7	2.4
Heterozygous 6	1.4	1.8	2.0	2.1	

S4 Table. Raw data set for Fig. 4B. Change in body weight (%) of MMA mice treated with vehicle or three dose levels of mLB-001 on PND 1

Animal	Treatment	Age (month)												
		**0.0**	**0.5**	**0.9**	**1.5**	**2.0**	**2.5**	**3.0**	**3.5**	**4.0**	**4.5**	**5.0**	**5.5**	**6.0**
V1	Vehicle	10.6	47.9	64.8	77.5	93.0	96.5	100.0	98.6	98.6	88.0	96.5		
V2	Vehicle	8.6	50.8	69.7	80.5	97.8	93.5	100.0	85.9	85.9	80.0	82.2	84.3	80.0
V3	Vehicle	9.8	54.6	79.8	85.8	96.7	97.8	100.0	86.9	88.5	88.5	86.3	86.3	84.7
V4	Vehicle	7.4	50.0	69.1	79.8	93.1	87.8	100.0	75.0	79.3	77.1	76.6	76.6	76.1
V5	Vehicle	10.3	53.4	73.6	79.9	96.6	97.1	100.0	79.9	71.8				
V6	Vehicle	9.5	49.7	66.5	76.5	87.2	91.6	100.0	86.0	72.6	63.7	70.4	68.2	70.9
V7	Vehicle	9.5	50.8	70.9	81.0	89.4	93.9	100.0	92.2	92.2				
V8	Vehicle	9.6	56.9	70.1	79.6	91.6	94.0	100.0	83.8	86.2	82.0			
V9	Vehicle	10.8	51.8	69.8	87.1	96.4	97.8	100.0	94.2					
V10	Vehicle	8.0	47.2	72.4	81.6	90.8	96.3	100.0	80.4	84.0	76.1			
V11	Vehicle	6.5	42.5	58.9	59.3	78.5	84.6	100.0	80.8	65.9				
V12	Vehicle	7.5	41.4	55.5	72.2	89.9	93.0	100.0	89.4	91.2	76.7			
V13	Vehicle	8.8	54.1	62.4	71.2	87.6	93.5	100.0	103.5	97.6	88.8	88.2	84.7	88.2
V14	Vehicle	9.3	40.4	58.4	81.4	84.5	98.8	100.0	99.4	90.7	85.1			
L1	2.5x10^{13} vg/kg	7.7	45.9	70.7	77.3	93.9	96.1	100.0	87.8	90.6	91.2	98.9	101.1	109.9
L2	2.5x10^{13} vg/kg	8.5	56.4	76.4	81.8	89.7	95.8	100.0	95.8	98.2	95.8	90.3	115.2	128.5
L3	2.5x10^{13} vg/kg	11.4	51.4	76.0	77.1	93.7	94.3	100.0	90.3	103.4	98.3	101.7	109.1	114.9
L4	2.5x10^{13} vg/kg	13.2	54.3	78.8	85.4	104.6	94.7	100.0	98.7	104.0	103.3	106.0	111.3	122.5
L5	2.5x10^{13} vg/kg	8.2	47.3	67.6	78.3	88.4	91.8	100.0	90.3	83.1	87.9	84.1	85.5	86.5
L6	2.5x10^{13} vg/kg	8.8	45.3	61.8	77.1	81.2	90.0	100.0	75.9	70.6	71.8	72.9	72.4	70.6
L7	2.5x10^{13} vg/kg	9.6	59.6	86.5	83.7	102.8	96.6	100.0	88.8	110.7	91.6	94.4	98.3	96.6
L8	2.5x10^{13} vg/kg	8.4	60.9	86.6	82.7	92.7	92.2	100.0	87.2					
L9	2.5x10^{13} vg/kg	9.0	58.0	74.5	79.0	91.5	92.0	100.0	86.5	96.5	83.0	82.0	82.0	79.0
L10	2.5x10^{13} vg/kg	11.3	49.1	73.6	77.4	90.6	92.5	100.0	86.2	89.9	81.1	83.0	83.6	83.6
L11	2.5x10^{13} vg/kg	8.3	51.2	72.0	75.6	86.3	98.2	100.0	95.2	92.9	93.5	95.2	97.0	109.5
L12	2.5x10^{13} vg/kg	7.0	45.3	60.7	74.6	80.6	94.5	100.0	95.5	101.5	83.1	81.6		
L13	2.5x10^{13} vg/kg	7.3	37.9	57.1	63.0	76.3	78.1	100.0	97.7	99.5	85.8	84.0	90.4	84.9
L14	2.5x10^{13} vg/kg	8.5	43.2	69.3	78.4	87.9	96.0	100.0	103.0	103.5	92.5	86.9	85.4	85.9
L15	2.5x10^{13} vg/kg	9.5	47.0	59.5	76.8	88.7	99.4	100.0	86.9	112.5	110.7	117.3	130.4	132.7
L16	2.5x10^{13} vg/kg	9.8	41.5	65.6	69.4	88.0	91.3	100.0	100.0	97.3	84.7	84.2	77.0	
M1	5x10^{13} vg/kg	9.2	56.7	71.6	77.3	87.2	96.5	100.0	96.5	114.9	112.1	111.3	120.6	122.0
M2	5x10^{13} vg/kg	7.9	53.4	72.3	82.7	97.4	97.4	100.0	87.4	89.5	83.8	89.5	88.5	90.6
M3	5x10^{13} vg/kg	7.7	50.8	73.5	82.3	95.0	99.4	100.0	84.0	84.5	91.7	96.1	98.3	102.8
M4	5x10^{13} vg/kg	8.8	49.7	72.9	80.1	88.4	96.1	100.0	86.7	82.3	89.5	96.7	101.1	115.5
M5	5x10^{13} vg/kg	12.5	39.8	58.5	77.8	93.2	94.3	100.0	85.8	90.3	85.8	85.2	87.5	92.6
M6	5x10^{13} vg/kg	11.8	51.3	79.6	86.8	93.4	96.7	100.0	93.4	95.4	89.5	83.6		
M7	5x10^{13} vg/kg	10.8	47.1	69.1	81.9	89.2	95.6	100.0	94.1	84.8	79.9	81.9	80.4	89.2
M8	5x10^{13} vg/kg	11.5	51.1	73.6	81.0	89.1	98.3	100.0	98.9	101.7	82.2	81.0	79.9	83.3
M9	5x10^{13} vg/kg	5.9	39.4	56.6	61.5	80.5	89.6	100.0	95.0	93.7	95.5	112.2	118.1	131.7
M10	5x10^{13} vg/kg	7.7	47.5	69.1	70.2	80.1	86.7	100.0	108.3	101.7	91.2			

Animal	Treatment	Age (month)												
		**0.0**	**0.5**	**0.9**	**1.5**	**2.0**	**2.5**	**3.0**	**3.5**	**4.0**	**4.5**	**5.0**	**5.5**	**6.0**
M11	$5x10^{13}$ vg/kg	7.8	42.7	61.7	76.7	94.2	101.0	100.0	89.3	85.0	77.7	78.2	77.2	79.1
M12	$5x10^{13}$ vg/kg	10.9	46.2	80.8	68.6	80.8	94.2	100.0	98.1	105.8	97.4	94.2	103.8	130.8
M13	$5x10^{13}$ vg/kg	9.6	37.2	62.8	67.0	76.1	93.6	100.0	87.2	90.4	83.5	79.3	76.6	84.6
M14	$5x10^{13}$ vg/kg	8.5	36.7	62.8	68.6	71.8	92.6	100.0	85.1	106.4	95.7	93.1	93.1	116.5
M15	$5x10^{13}$ vg/kg	8.3	38.1	61.9	65.5	77.4	89.9	100.0	85.1	89.9	78.6	78.6	84.5	96.4
M16	$5x10^{13}$ vg/kg	11.0	45.7	70.5	78.6	81.4	91.9	100.0	107.1	102.4	87.1	81.4		
H1	$1x10^{14}$ vg/kg	9.9	52.8	81.0	88.0	94.4	95.1	100.0	97.2	109.2	94.4	104.2	107.0	117.6
H2	$1x10^{14}$ vg/kg	10.1	63.1	75.0	80.4	96.4	97.0	100.0	96.4	91.7	92.9	95.8	97.6	106.0
H3	$1x10^{14}$ vg/kg	9.0	56.5	70.6	74.0	89.8	92.1	100.0	96.6	103.4	100.0	106.2	105.6	114.7
H4	$1x10^{14}$ vg/kg	8.2	38.3	53.6	79.6	92.3	92.3	100.0	100.5	107.7	99.5	104.1	112.2	116.3
H5	$1x10^{14}$ vg/kg	8.7	50.5	68.5	78.8	94.0	97.8	100.0	95.1	107.6	112.0	116.3	122.8	129.3
H6	$1x10^{14}$ vg/kg	14.8	52.3	66.4	77.9	94.0	100.0	100.0	87.2	87.9	89.3	97.3	104.0	115.4
H7	$1x10^{14}$ vg/kg	14.2	45.7	61.1	71.0	89.5	95.1	100.0	103.7	103.1	103.7	104.3	106.8	103.7
H8	$1x10^{14}$ vg/kg	9.9	46.6	60.2	65.8	72.0	86.3	100.0	93.8	100.6	95.0	108.7	129.2	136.6
H9	$1x10^{14}$ vg/kg	8.7	48.8	61.8	76.3	90.3	94.7	100.0	103.9	102.9	85.5	87.9	88.9	95.7
H10	$1x10^{14}$ vg/kg	8.5	37.6	51.2	71.8	84.5	93.0	100.0	106.1	107.5	88.3	83.1	86.4	93.9
H11	$1x10^{14}$ vg/kg	11.6	45.1	61.6	75.0	86.0	101.2	100.0	94.5	85.4	95.1	101.8	109.8	109.8
H12	$1x10^{14}$ vg/kg	11.7	41.0	56.9	71.3	80.3	89.4	100.0	90.4	100.0	87.2			
H13	$1x10^{14}$ vg/kg	14.6	52.1	63.9	84.7	90.3	96.5	100.0	100.7	100.0	85.4	89.6	102.1	115.3
H14	$1x10^{14}$ vg/kg	12.7	49.2	71.3	75.1	89.0	94.5	100.0	86.7	95.0	98.3	109.9	130.4	145.3

S5 Table. Raw data set for Fig. 4C. Plasma ALB-2A levels (µg/mL) in heterozygous and MMA mice treated with three dose levels of mLB-001 on PND 1

Animal	Genotype	Treatment	Age (month)					
			1	2	3	4	5	6
HE L1	Heterozygous	2.5x10^{13} vg/kg	0.4	0.5	0.5	0.6		
HE L2	Heterozygous	2.5x10^{13} vg/kg	0.3	0.4	0.4	0.5		
HE L3	Heterozygous	2.5x10^{13} vg/kg	0.5	0.7	0.6	0.7		
HE L4	Heterozygous	2.5x10^{13} vg/kg		0.5	0.6	0.6	0.5	0.6
HE L5	Heterozygous	2.5x10^{13} vg/kg	0.4	0.5	0.6	0.7	0.6	0.6
HE L6	Heterozygous	2.5x10^{13} vg/kg	1.3	0.5	0.5	0.5	0.5	0.5
HE L7	Heterozygous	2.5x10^{13} vg/kg	1.1	1.9	1.9	2.0		
HE L8	Heterozygous	2.5x10^{13} vg/kg	0.8	0.9	1.0	1.2		
HE L9	Heterozygous	2.5x10^{13} vg/kg	0.7	0.7	0.9	1.1		
HE L10	Heterozygous	2.5x10^{13} vg/kg	0.4	0.3	0.6	0.6	0.6	0.3
HE L11	Heterozygous	2.5x10^{13} vg/kg	0.9	1.1	1.4	1.3		
HE M1	Heterozygous	5x10^{13} vg/kg	0.9	1.0	0.9	0.8	1.1	
HE M2	Heterozygous	5x10^{13} vg/kg	0.8	1.0	0.9		1.0	
HE M3	Heterozygous	5x10^{13} vg/kg	0.8	1.3	1.2	1.5	1.4	
HE M4	Heterozygous	5x10^{13} vg/kg	1.3	1.1	1.7	2.0	2.0	2.3
HE M5	Heterozygous	5x10^{13} vg/kg	0.9	1.2	1.2	1.3		
HE M6	Heterozygous	5x10^{13} vg/kg	1.0	1.2	1.7	1.4		
HE M7	Heterozygous	5x10^{13} vg/kg		1.2	1.2	1.4	1.4	1.3
HE M8	Heterozygous	5x10^{13} vg/kg	1.2	1.4	1.5	1.4	1.6	2.1
HE M9	Heterozygous	5x10^{13} vg/kg	1.3	1.8	2.3	2.2		
HE M10	Heterozygous	5x10^{13} vg/kg	0.6	1.2	1.2	1.1	1.1	
HE M11	Heterozygous	5x10^{13} vg/kg		1.7	1.5	1.4	1.6	
HE M12	Heterozygous	5x10^{13} vg/kg		1.3	1.5	1.4	1.6	
HE M13	Heterozygous	5x10^{13} vg/kg		1.2		1.5	1.3	1.2
HE M14	Heterozygous	5x10^{13} vg/kg	0.5	0.7	0.9	0.8		
HE M15	Heterozygous	5x10^{13} vg/kg	0.7	1.0	1.1	1.2		
HE M16	Heterozygous	5x10^{13} vg/kg	0.5	0.8	1.0	1.0		
HE H1	Heterozygous	1x10^{14} vg/kg	1.5	0.9	1.7	1.9	2.2	2.0
HE H2	Heterozygous	1x10^{14} vg/kg	0.8	1.7	1.0	1.1	1.2	1.3
HE H3	Heterozygous	1x10^{14} vg/kg		1.5	1.5	1.6	1.6	1.9
HE H4	Heterozygous	1x10^{14} vg/kg	1.3	1.8	2.1	2.1	2.2	2.6
HE H5	Heterozygous	1x10^{14} vg/kg	1.2	2.2		2.3	2.0	2.2
HE H6	Heterozygous	1x10^{14} vg/kg	2.8	2.5	2.6	3.4	1.9	1.8
HE H7	Heterozygous	1x10^{14} vg/kg	3.8	4.7	4.6	6.1		
HE H8	Heterozygous	1x10^{14} vg/kg	4.2	4.5	5.6	7.2		
HE H9	Heterozygous	1x10^{14} vg/kg	2.4	2.5	2.8	3.1		
HE H10	Heterozygous	1x10^{14} vg/kg	2.1	3.8	4.1	3.4	3.8	
HE H11	Heterozygous	1x10^{14} vg/kg	2.3	4.1	3.8	3.5	3.7	
HE H12	Heterozygous	1x10^{14} vg/kg	2.4	4.0	4.8	4.2	4.3	
HE H13	Heterozygous	1x10^{14} vg/kg		2.9	3.6	3.2	3.6	
HE H14	Heterozygous	1x10^{14} vg/kg		2.8	3.6	3.6	3.7	
HE H15	Heterozygous	1x10^{14} vg/kg	2.1	3.1	3.8	3.1	3.7	
HE H16	Heterozygous	1x10^{14} vg/kg	3.2	3.4	3.5			
MMA L1	MMA	2.5x10^{13} vg/kg	1.0					
MMA L2	MMA	2.5x10^{13} vg/kg	0.9	2.7	5.9	43.5	20.9	31.6
MMA L3	MMA	2.5x10^{13} vg/kg	0.9					
MMA L4	MMA	2.5x10^{13} vg/kg	1.3	8.5	29.3	162.9	482.2	743.2
MMA L5	MMA	2.5x10^{13} vg/kg	0.8	4.4	7.2	27.9	63.5	92.8
MMA L6	MMA	2.5x10^{13} vg/kg	0.8	5.8	10.4			
MMA L7	MMA	2.5x10^{13} vg/kg	1.0	4.8	7.3	23.1	13.5	15.1
MMA L8	MMA	2.5x10^{13} vg/kg	0.7	3.2	7.6	40.9	48.4	137.1
MMA L9	MMA	2.5x10^{13} vg/kg	0.8	7.4	26.3	182.2	154.9	420.8
MMA L10	MMA	2.5x10^{13} vg/kg	0.7	4.9	6.8	43.4	50.5	148.4
MMA L11	MMA	2.5x10^{13} vg/kg	0.6	3.5	4.4	18.3	44.3	

Animal	Genotype	Treatment	Age (month)					
			1	2	3	4	5	6
MMA L12	MMA	$2.5x10^{13}$ vg/kg		10.2				
MMA L13	MMA	$2.5x10^{13}$ vg/kg	0.7					
MMA L14	MMA	$2.5x10^{13}$ vg/kg	0.9	9.1	13.0	86.2	136.4	281.6
MMA L15	MMA	$2.5x10^{13}$ vg/kg	0.5	5.3	8.8	46.1	66.1	144.5
MMA L16	MMA	$2.5x10^{13}$ vg/kg	0.7	5.1	13.6	39.1	62.5	119.5
MMA L17	MMA	$2.5x10^{13}$ vg/kg	1.5	4.6	12.0	36.3	56.4	129.4
MMA L18	MMA	$2.5x10^{13}$ vg/kg	1.1	3.4	6.6	15.5	28.8	34.3
MMA L19	MMA	$2.5x10^{13}$ vg/kg	0.9	3.9	4.6	10.5	18.7	
MMA L20	MMA	$2.5x10^{13}$ vg/kg	1.2	4.4	8.3	32.6	48.1	95.6
MMA M1	MMA	$5x10^{13}$ vg/kg	4.3	9.9	10.1	63.2		
MMA M2	MMA	$5x10^{13}$ vg/kg	1.8					
MMA M3	MMA	$5x10^{13}$ vg/kg	1.4	16.9	26.5	174.8	428.9	846.4
MMA M4	MMA	$5x10^{13}$ vg/kg	3.9					
MMA M5	MMA	$5x10^{13}$ vg/kg	1.6	9.7	17.9	96.5	223.7	308.6
MMA M6	MMA	$5x10^{13}$ vg/kg	1.3	8.2				
MMA M7	MMA	$5x10^{13}$ vg/kg	1.8	12.0	27.9	96.2	108.6	
MMA M8	MMA	$5x10^{13}$ vg/kg	1.2	7.9	19.1	99.5	220.4	440.1
MMA M9	MMA	$5x10^{13}$ vg/kg	1.6	10.2	23.7	82.6	155.3	271.9
MMA M10	MMA	$5x10^{13}$ vg/kg	2.0	12.0	21.5	98.6	175.5	428.5
MMA M11	MMA	$5x10^{13}$ vg/kg	2.6	33.5	89.1	333.6	714.8	872.2
MMA M12	MMA	$5x10^{13}$ vg/kg	1.5					
MMA M13	MMA	$5x10^{13}$ vg/kg	1.1	4.9	8.5	33.3	63.0	119.5
MMA M14	MMA	$5x10^{13}$ vg/kg	1.1	5.6	10.4	18.5	27.7	66.2
MMA M15	MMA	$5x10^{13}$ vg/kg	1.4	6.8	11.5	33.8	54.9	
MMA M16	MMA	$5x10^{13}$ vg/kg	1.0	4.5	8.3	40.2	56.1	119.8
MMA M17	MMA	$5x10^{13}$ vg/kg	1.6	9.9	19.8	96.6	91.0	205.4
MMA M18	MMA	$5x10^{13}$ vg/kg	2.4	6.6		41.0	64.6	131.3
MMA M19	MMA	$5x10^{13}$ vg/kg	2.7	12.7	54.7	243.2	261.0	539.2
MMA M20	MMA	$5x10^{13}$ vg/kg	0.6	3.2	6.6	52.3	86.5	172.3
MMA M21	MMA	$5x10^{13}$ vg/kg	1.3	8.3	16.3	37.9		
MMA M22	MMA	$5x10^{13}$ vg/kg	0.8	6.8	25.8			
MMA H1	MMA	$1x10^{14}$ vg/kg	4.3	21.7	60.9	332.6	675.9	990.7
MMA H2	MMA	$1x10^{14}$ vg/kg	2.4	26.0	61.6	285.6	519.9	
MMA H3	MMA	$1x10^{14}$ vg/kg		20.2	31.5	153.8	277.2	503.4
MMA H4	MMA	$1x10^{14}$ vg/kg		20.0		270.6	434.4	591.6
MMA H5	MMA	$1x10^{14}$ vg/kg	1.8	42.5				
MMA H6	MMA	$1x10^{14}$ vg/kg		22.7	29.8	108.4		
MMA H7	MMA	$1x10^{14}$ vg/kg	4.0	19.3	111.7	447.4	677.1	1020.8
MMA H8	MMA	$1x10^{14}$ vg/kg	5.0	15.0	53.2	173.1	265.2	333.5
MMA H9	MMA	$1x10^{14}$ vg/kg	4.0					
MMA H10	MMA	$1x10^{14}$ vg/kg			45.8	162.6	222.3	485.9
MMA H11	MMA	$1x10^{14}$ vg/kg	2.7					
MMA H12	MMA	$1x10^{14}$ vg/kg	3.3	28.2	81.2	279.5	417.3	723.7
MMA H13	MMA	$1x10^{14}$ vg/kg	3.4	18.8	27.6	143.6	298.5	429.8
MMA H14	MMA	$1x10^{14}$ vg/kg	4.2	21.6	24.3	142.5	295.4	436.3
MMA H15	MMA	$1x10^{14}$ vg/kg	3.3	21.0	29.1	90.4	186.8	264.3
MMA H16	MMA	$1x10^{14}$ vg/kg						
MMA H17	MMA	$1x10^{14}$ vg/kg	3.0	28.7	80.8	276.5	662.6	791.0
MMA H18	MMA	$1x10^{14}$ vg/kg	5.0	29.5	40.4	125.9	371.4	379.2
MMA H19	MMA	$1x10^{14}$ vg/kg	2.5	10.6	27.2	106.4		

S6 Table. Raw data set for Fig. 5C. Change in body weight (%) of MMA mice treated with vehicle or 5×10^{13} vg/kg mLB-001 at 8 weeks of age

Animal	Treatment	Age (month)						
		**5.0**	**5.6**	**6.2**	**6.5**	**6.9**	**7.3**	**8.0**
V1	Vehicle	100.0	92.8	90.3	83.1	87.3	79.3	83.5
V2	Vehicle	100.0	85.4	91.4	79.5			
V3	Vehicle	100.0	91.5	98.1	68.2			
V4	Vehicle	100.0	99.1	97.4	74.1	71.6	68.5	72.4
V5	Vehicle	100.0	100.4	92.0	71.6	76.4	71.6	
V6	Vehicle	100.0	89.0	91.7	74.6	74.6	72.3	
T1	5x10^{13} vg/kg	100.0	99.5	113.4	103.0	106.4	113.9	109.9
T2	5x10^{13} vg/kg	100.0	93.1	107.9	104.0	110.4	107.9	112.9
T3	5x10^{13} vg/kg	100.0	103.9	106.9	90.1	92.6	95.1	105.9
T4	5x10^{13} vg/kg	100.0	86.4	93.5	90.0	96.8	103.2	109.3
T5	5x10^{13} vg/kg	100.0	91.9	94.4				
T6	5x10^{13} vg/kg	100.0	100.4	104.4	92.0	100.0	97.5	101.1
T7	5x10^{13} vg/kg	100.0	94.8	105.5	84.9	88.6	90.8	101.8

S7 Table. Raw data set for Fig. 5D. Circulating methylmalonic acid levels (µM) in MMA mice treated with vehicle or 5×10^{13} vg/kg mLB-001 at 8 weeks of age

Animal	Treatment	Age (month)							
		1	2	3	4	5	6	6.8	8
V1	Vehicle	906	1440	452	481	629	906	781	1135
V2	Vehicle	783	885	760	870	1087	1284	2120	
V3	Vehicle	614	527	422	881	737	1638	1336	
V4	Vehicle	525	987	467	436	705	916	1565	1530
V5	Vehicle	1714	858	427	392	777	958		
V6	Vehicle	961	707	395	416	864	708		
V7	Vehicle	706	1368	1130					
T1	$5x10^{13}$ vg/kg	1139	784	606	336	730	702	643	797
T2	$5x10^{13}$ vg/kg	893	853	487	315	427	262	294	595
T3	$5x10^{13}$ vg/kg	1219	716	465	347	443	414	504	417
T4	$5x10^{13}$ vg/kg	1141	670	296	413	405	710	784	526
T5	$5x10^{13}$ vg/kg	1704	618	461	501	698	561		
T6	$5x10^{13}$ vg/kg	869	784	416	334	402	318	225	500
T7	$5x10^{13}$ vg/kg	1115	916	365	465	507	539	466	412

S8 Table. Raw data set for Fig. 5E. Plasma ALB-2A levels (µg/mL) in heterozygous and MMA mice treated with 5×10^{13} vg/kg mLB-001 at 8 weeks of age

**Animal**	**Age (month)**					
	**3**	**4**	**5**	**6**	**7**	**8**
MMA 1	10.7	31.5	41.4	104.3	156.3	315.6
MMA 2	5.1	17.2	34.2	218.1	154.0	221.6
MMA 3	11.7	26.5	54.1	168.8	204.9	308.3
MMA 4	10.6	17.7	26.9	66.0	121.5	214.2
MMA 5	1.4	3.4	5.0	13.2		
MMA 6	18.7	69.8	161.6	296.6	379.0	341.5
MMA 7	10.2	18.5	50.8	232.8	201.6	298.3
Heterozygous 1	0.7	0.5	0.7	0.7		0.8
Heterozygous 2	0.8	0.8	0.8	0.8		1.3
Heterozygous 3	1.4	1.6	1.6	1.5		
